# Supplementary material for: Nanoscale Mapping of the Subcellular Glycosylation Landscape
Source: Adv Sci (Weinh). 2025 Nov 10;13(11):e06731. doi: 10.1002/advs.202506731 (PMC12931256; doi:10.1002/advs.202506731)
Supplement: Supplementary file 2 — Supporting Information [file ADVS-13-e06731-s001.docx]

# Supplementary Figures


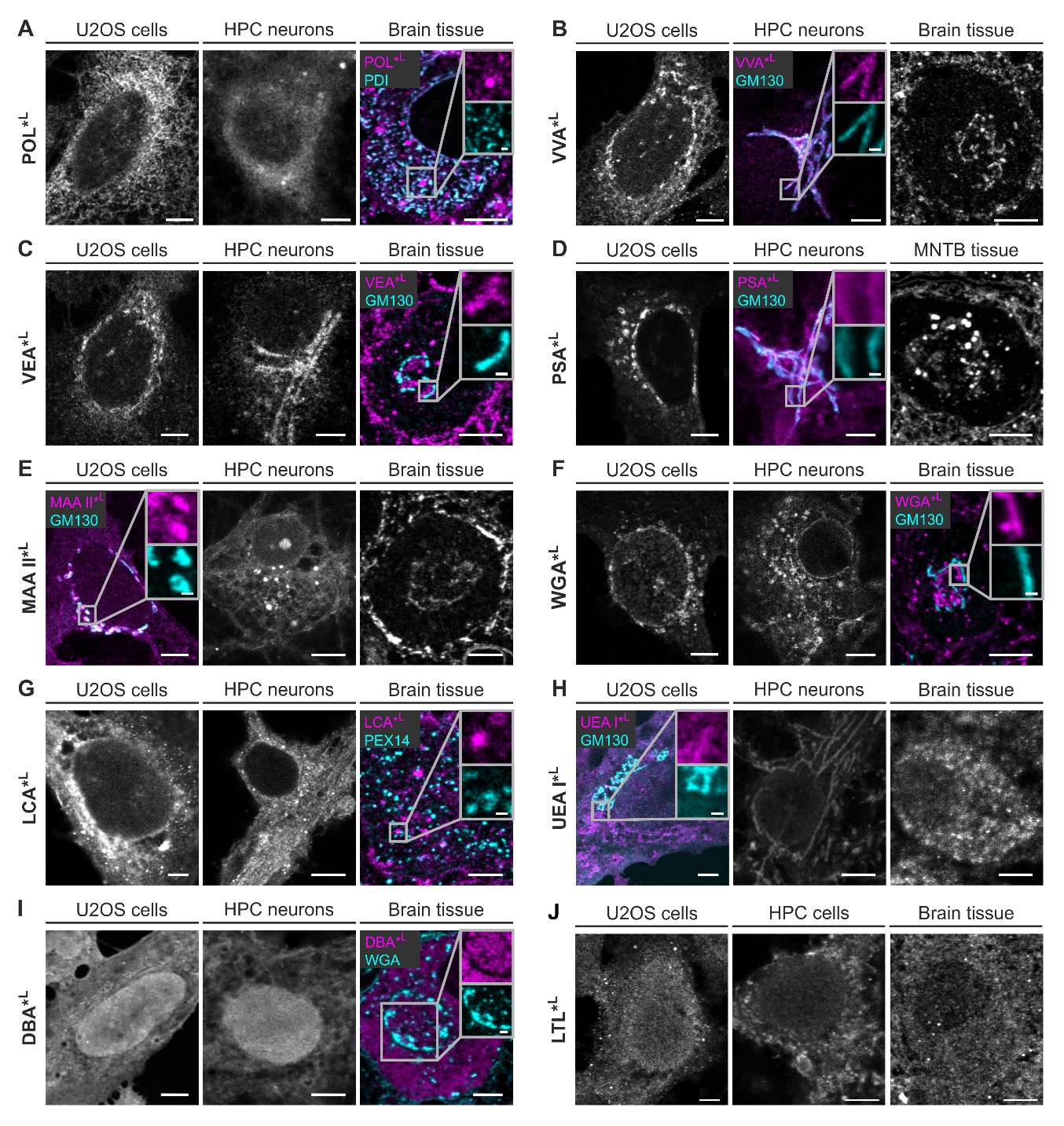


Figure S1. Extended results of lectin screening at the confocal microscope. Lectin staining in U2OS cells, hippocampal (HPC) neurons, and brain tissue. A) POL colocalizes with endoplasmic reticulum marker PDI. B-F) VVA, VEA, PSA, MAA II, and WGA exhibit staining at or close to the Golgi apparatus, as confirmed by spatial proximity with *cis*-Golgi marker GM130. G) LCA shows staining of small round clusters that do not colocalize with peroxisome marker PEX14. H) UEA I displays filamentous staining in hippocampal neurons and U2OS cells with only sparse signal in GM130-positive areas. No filamentous signal was observed in brain tissue. I) DBA shows abundant nuclear and cytosolic staining but is clearly absent in the Golgi area in principal cells of brain sections. J) LTL displays broad, partially clustered nucleocytoplasmic staining. Scale bars correspond to 5 µm in all overview images and 1 µm in the insets.


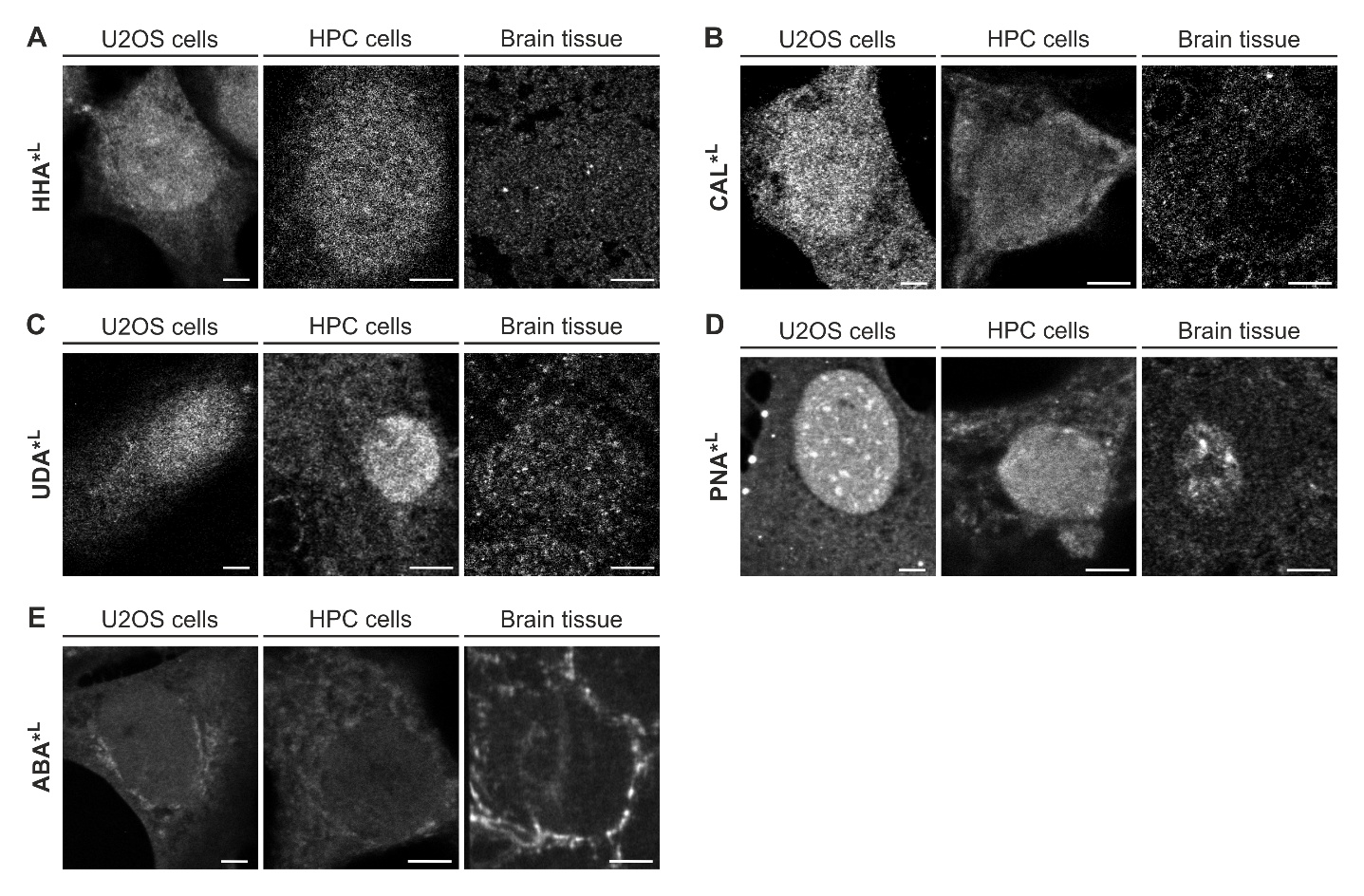


Figure S2**.** Lectins with weak signals or variable performance. A-C) HHA, CAL, and UDA showed diffuse cytoplasmic and nuclear staining in most cell/tissue models, often with low-intensity signals. D,E) Image panels represent the predominant staining patterns for PNA and ABA, which varied across experiments. Scale bars correspond to 5 µm.


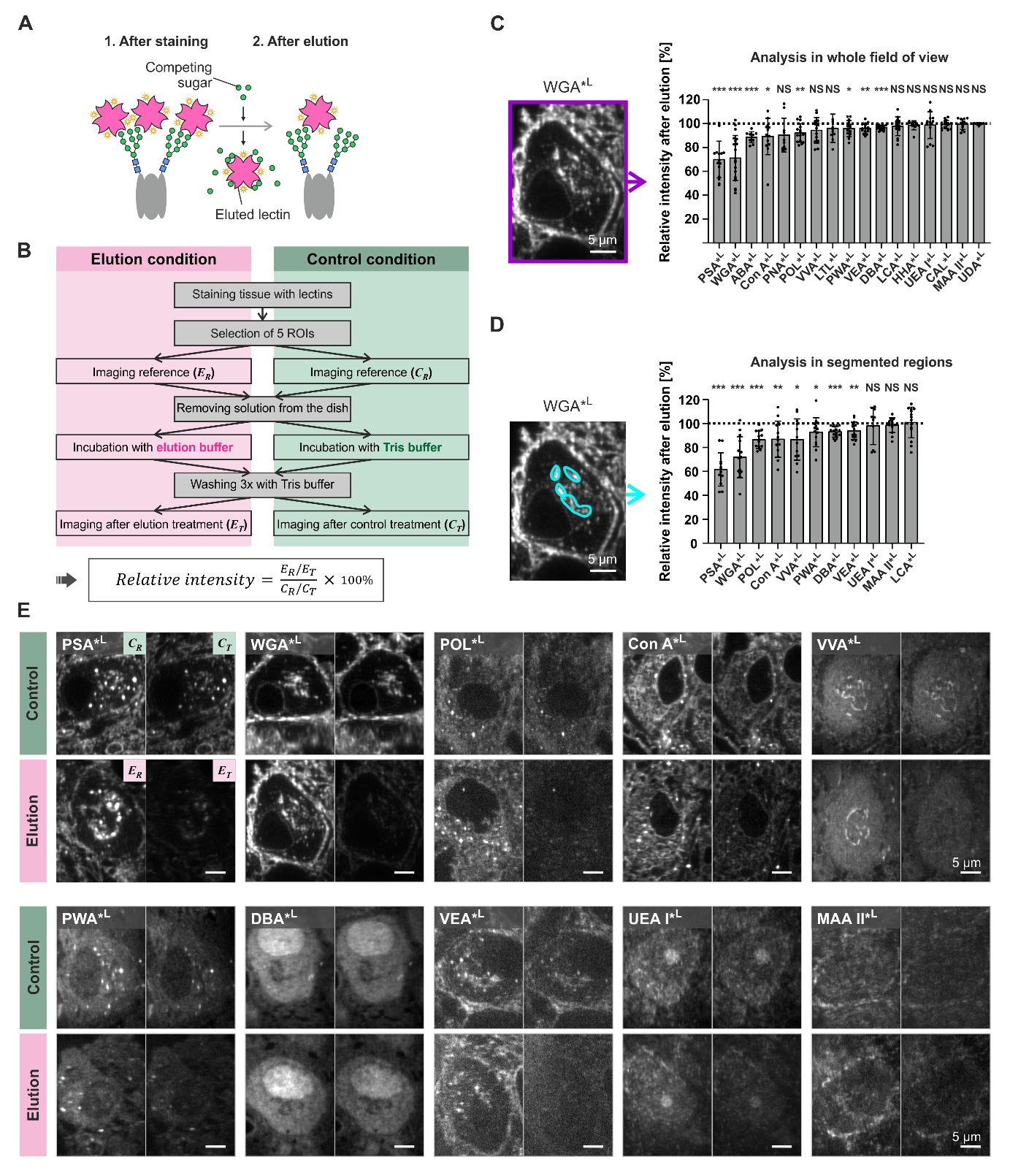


**Figure S3.** Characterization of lectin specificity by elution experiments. A) Elution experiments using sugars competing with carbohydrates recognized by the respective lectins were performed to characterize lectin specificity. Example illustrating elution of a mannose-binding lectin using mannosides. B) Experimental workflow for elution experiments. C-D) Signal intensity was either quantified in full-frame images containing whole cells (C) or in specifically segmented cell areas (D). Data represent mean intensity percentage ±SD from *n* = 5 to 15 analyzed principal cells per lectin. Asterisks denote statistical significance (***p ≤ 0.0001, **p ≤ 0.01, *p ≤ 0.05, NS = not significant; two-sided one sample t-test versus 100%). E) Images showing loss of signal after elution with competing sugars compared to control experiments visualizing the effect of bleaching and washing without sugar treatment (used as 100% reference).

**
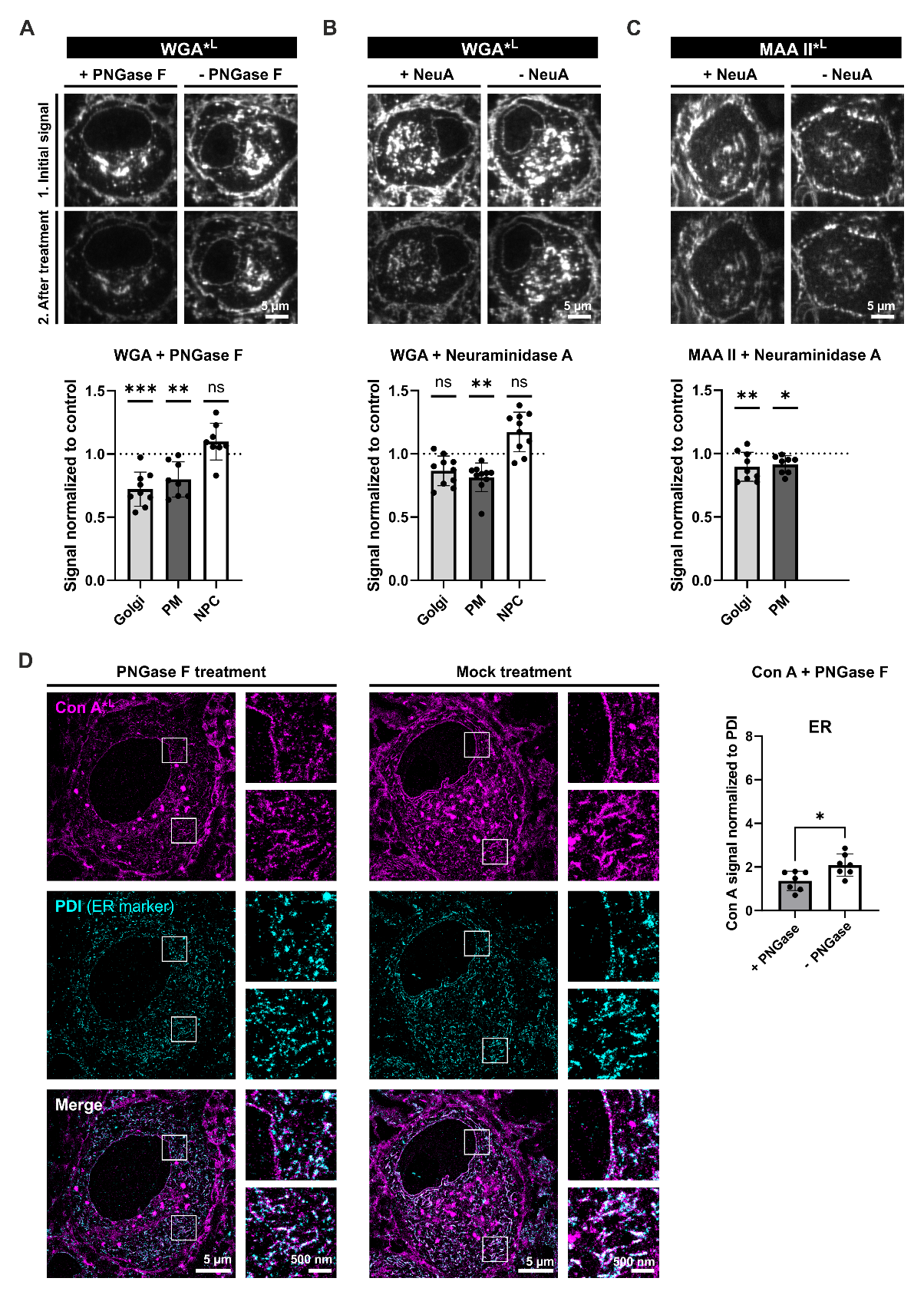
**

**Figure S4.** Glycosidase control experiments in fixed neuronal tissue. A-C) Wide-field images of lectin-stained neuronal tissue before and after PNGase F treatment (left) or mock treatment (right). A) Following PNGase F treatment, WGA signal is significantly reduced at the Golgi and plasma membrane regions enriched in N-glycans, and remains largely unchanged at the nuclear envelope, where WGA binds O-glycosylated nuclear pore proteins. B) Following Neuraminidase A treatment, WGA signal is reduced at the plasma membrane, where terminal sialic acids are abundant and accessible, while nuclear pores lacking sialylation remain unaffected. C) Significant reduction of MAA II labeling at the Golgi and plasma membrane region upon PNGase F treatment, consistent with MAA II binding to sialic acid-containing motifs. D) *d*STORM imaging of PNGase F- or mock-treated neuronal tissue followed by Con A staining revealed a significant reduction of Con A signal in endoplasmic reticulum segments after PNGase F treatment. A-D) Bars represent normalized means ±SD from 7 to 10 principal cells (7 ≤ *n* ≤ 10). Asterisks denote statistical significance (***p ≤ 0.001, **p ≤ 0.01, *p ≤ 0.05, ns = not significant; two-way ANOVA with Tukey’s post hoc test on raw data (A-C) or two-tailed, unpaired Student’s *t*-test (D)). ER, endoplasmic reticulum; PM, plasma membrane; NPC, nuclear pore complex; NeuA, Neuraminidase A.

**
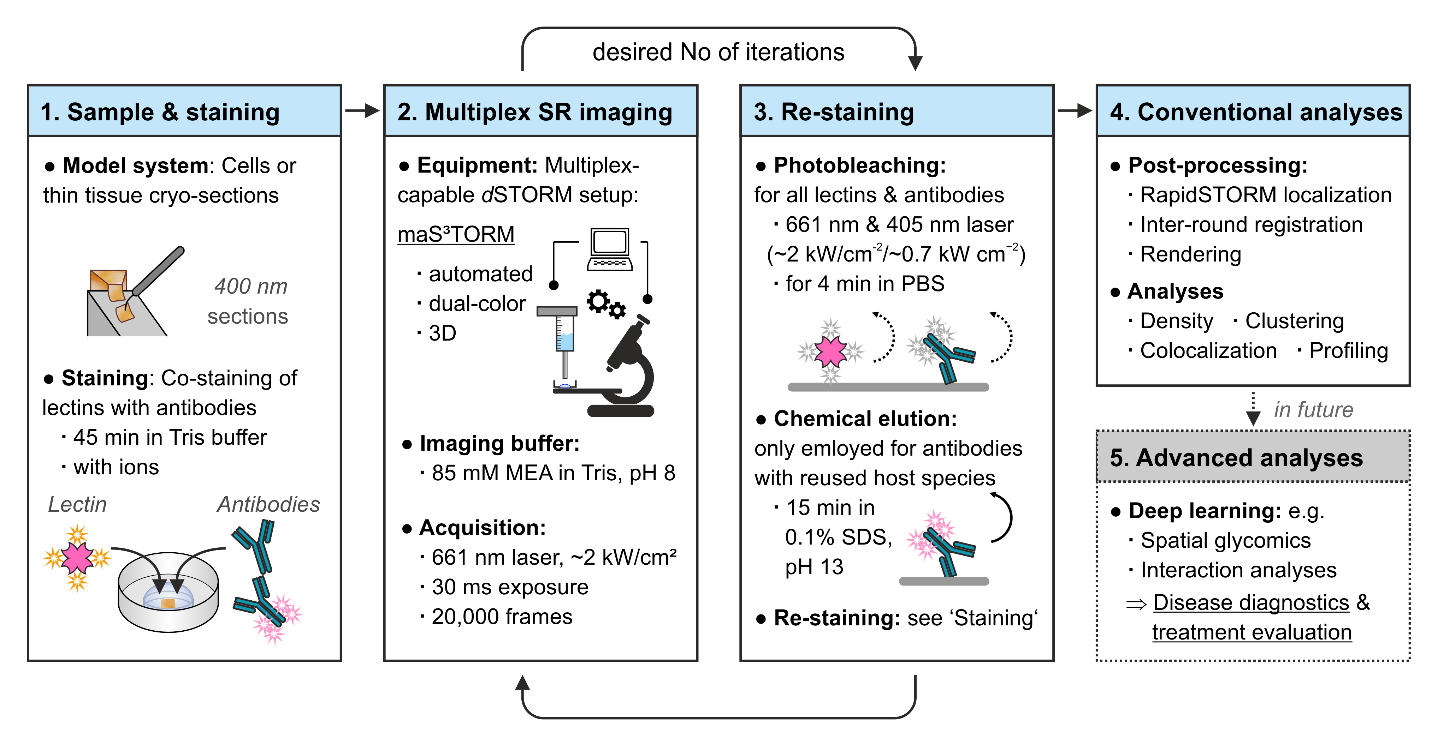
Figure S5.** Glyco-STORM concept. Strategy for Glyco-STORM imaging. (1) Staining of thin tissue or cells with lectins is realized in Tris buffer supplemented with ions and can be done together with classical immunohistochemistry stainings. (2) Automated 3D super-resolution (SR) imaging and re-staining of samples is realized using the maS³TORM setup that allows dual-channel acquisition of two spectrally close far-red fluorophores (in our case Alexa fluor 647 and CF647). Any other STORM setup and re-staining method can be used. (3) For re-staining, signals from lectins and antibodies imaged in the previous round are removed by photobleaching. Photobleaching causes quenching of all fluorophores and is expected to also result in photounbinding of a certain fraction of fluorophore-conjugated labels^[1,2]^ (dashed curved arrow). Chemical elution results in reliable removal of antibodies and is only realized after rounds preceding the usage of secondary antibodies against antibodies from host species already employed in previous rounds (solid curved arrow). (4) Data undergoes post-processing, including localization fitting, fiducial-aided registration of images from different rounds, and image rendering. Classical analysis methods suitable for super-resolution data can be applied. (5) Future directions: With the accumulation of sufficient data amenable to deep learning techniques, spatial glycomics, and interaction analyses will facilitate the comparison of glycosylation profiles of diseased versus healthy, or treated versus untreated samples.

**
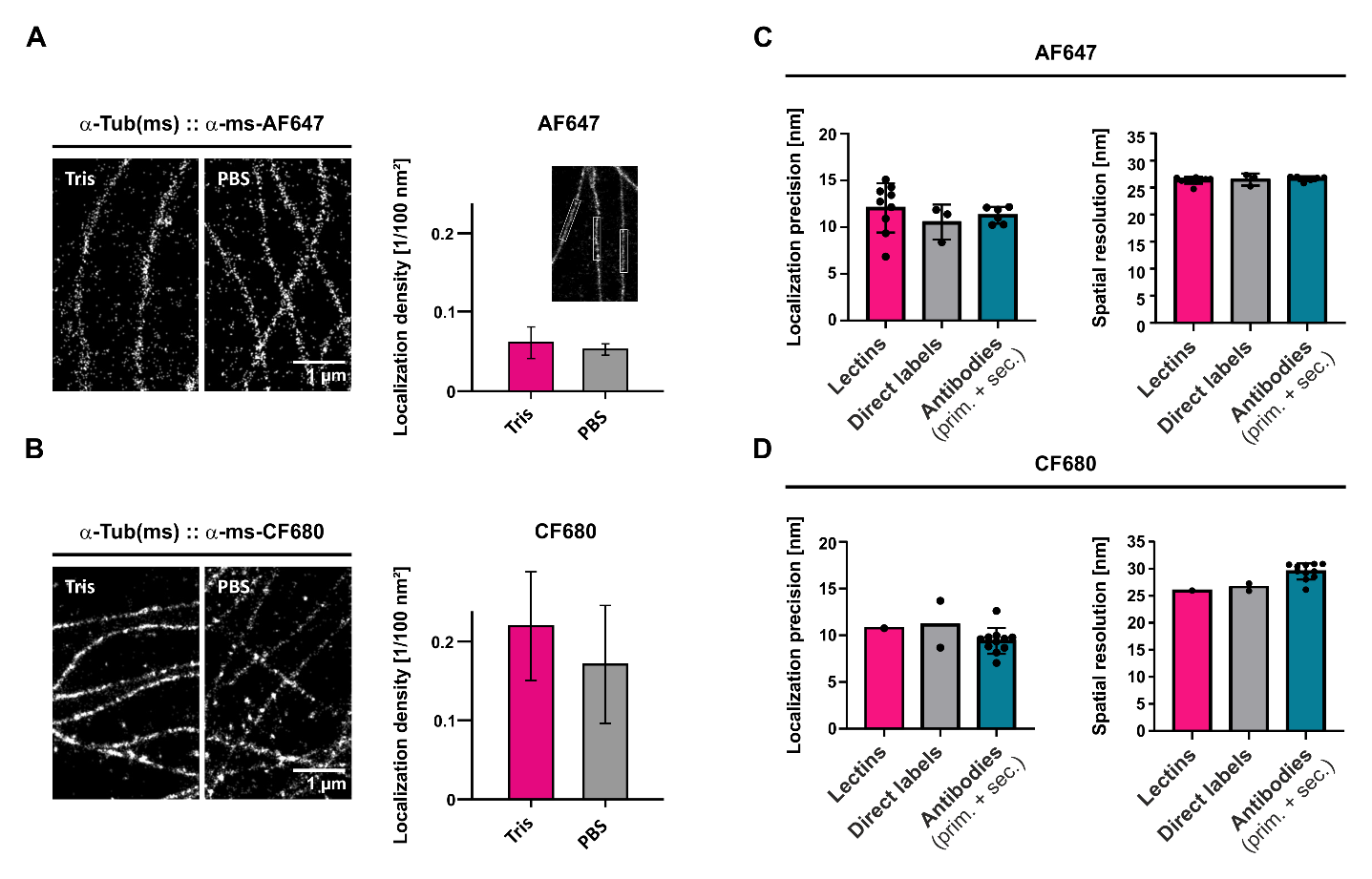
Figure S6.** Buffer optimization and evaluation of imaging precision for Glyco-STORM. A,B) Evaluation of Tris- versus PBS-based buffers for immunohistochemical staining and *d*STORM imaging. Tris-based buffers (staining: 50 mM Tris-HCl, 150 mM NaCl; imaging: 85 mM MEA in Tris, 35 mM KOH, pH 8) were used for Glyco-STORM to prevent ion precipitation in PBS (staining: PBS; imaging: 100 mM MEA in PBS, 20 mM KOH, pH 8). The effectiveness of Tris-based buffers was validated through classical immunohistochemical stainings, demonstrating similar *d*STORM image quality and localization density to PBS-based buffers, previously employed for maS³TORM^[2]^. Buffers were tested using the anti-α-Tubulin primary antibody from mouse and (A) the anti-mouse-AF647 or (B) the anti-mouse-CF680 secondary antibody. Bars represent mean density ±SD, *n* = 10 to 36 quantified microtubule segments (exemplified in the inset in A). C,D) Lectins result in localization precision (left panels) and spatial resolution (right panels) comparable to direct labels such as phalloidin or directly labeled primary antibodies and nanobodies. Each data point corresponds to a distinct label and is the average value of 3–5 analyzed *d*STORM images per label. Bar graphs represent mean ±SD.

**
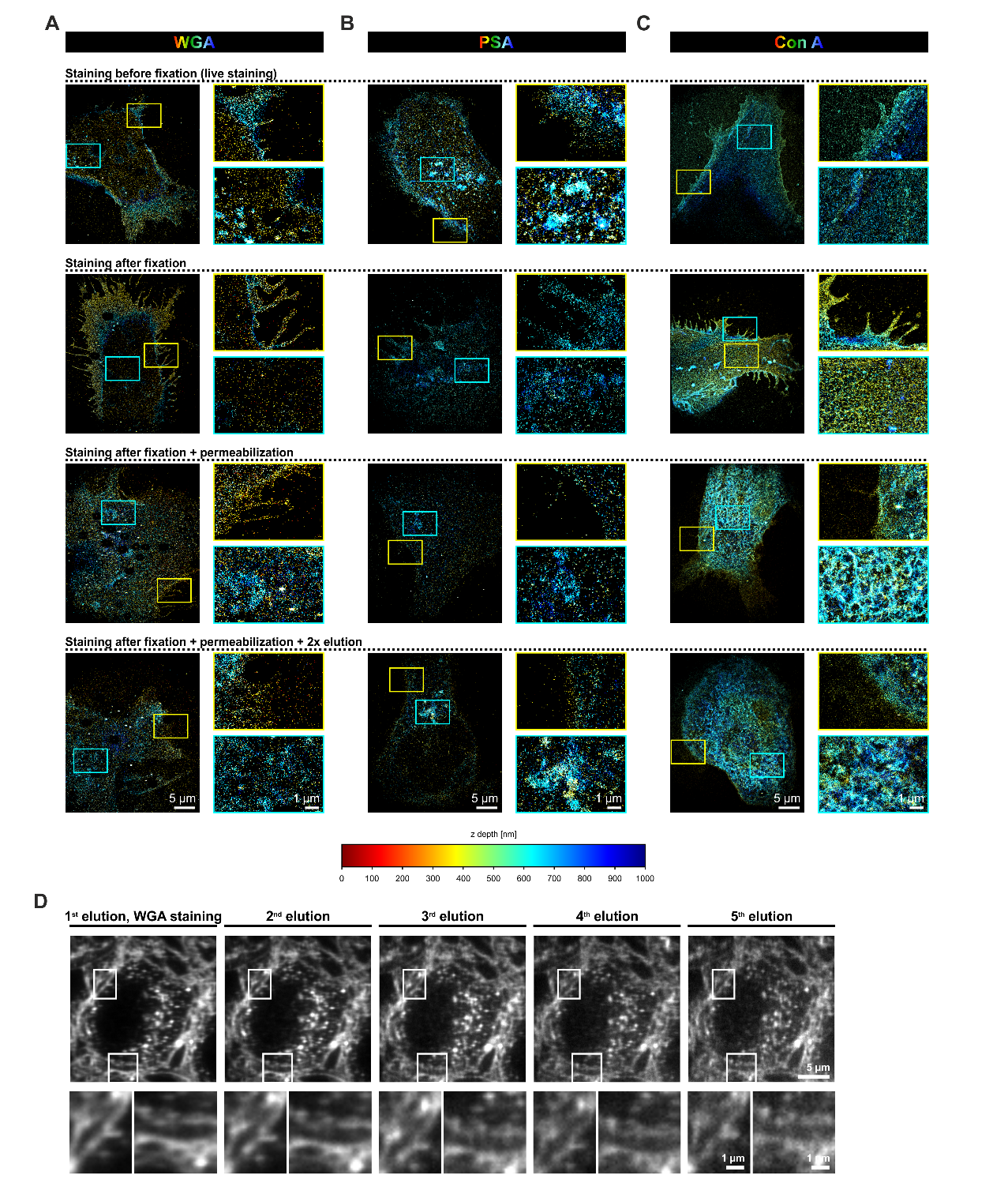
**

**Figure S7.** Effect of fixation, permeabilization, and elution on lectin staining. A-C) Color-coded 3D renderings of WGA (A), PSA (B), and Con A (C) lectin stainings in U2OS cells under different sample preparation conditions: before fixation (row 1), after fixation (row 2), after fixation and permeabilization (row 3), and after fixation, permeabilization, and elution (row 4). Magnified views highlight signals at the glycocalyx at the cell rim (yellow boxes) and in more central regions away from the rim (cyan boxes). Before fixation, staining is mainly restricted to the glycocalyx, with partial internalization observed during the 10 min incubation. After fixation, glycocalyx staining appears highly abundant. Following permeabilization, the signal shifts from the glycocalyx towards intracellular regions/organelles. After elution, the signal distribution remains comparable to the permeabilization-only condition. D) Wide-field images of WGA stainings of the calyx of Held synapse in neuronal tissue with two magnified regions of the presynapse, demonstrating that glycocalyx/plasma membrane signal remain preserved for many elution rounds. Images are intensity-normalized.

**
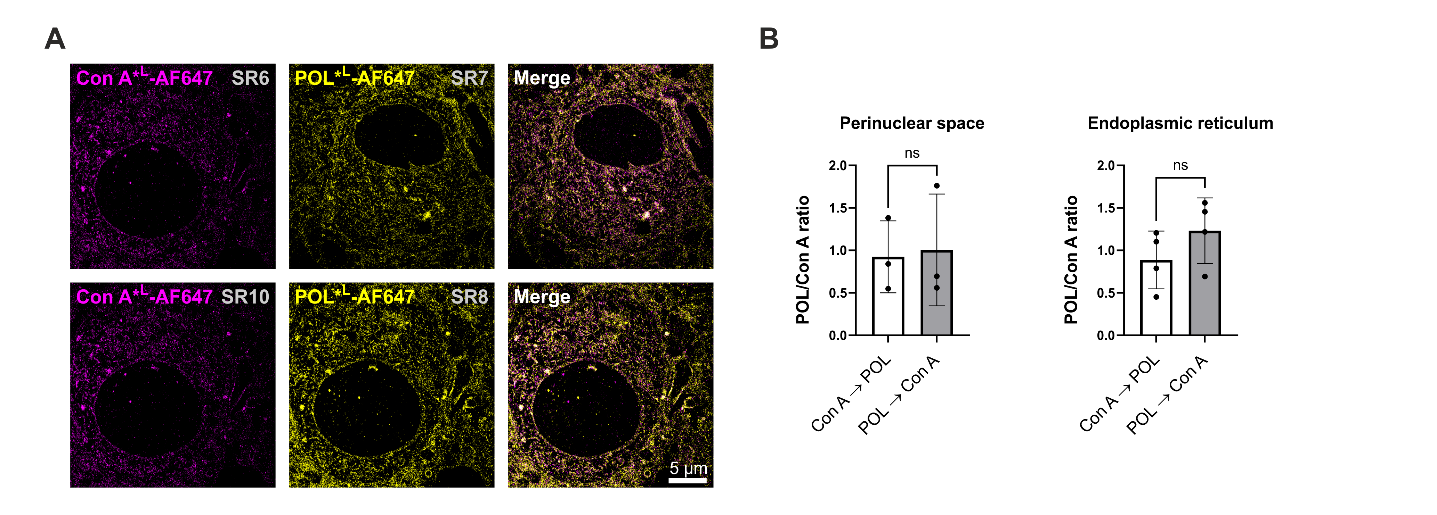
**

**Figure S8.** Competition between lectins of similar specificity. A) Sequential labeling with mannose-binding lectins Con A and POL with reversed application order. At the concentrations used in Glyco-STORM (Con A–AF647, BioWORLD, 0.05 mg/ml; Table S2), signals are comparable regardless of application sequence. SR, staining round. B) Analysis of perinuclear and endoplasmic reticulum regions shows no significant change in the POL/Con A ratio irrespective of staining sequence. Bars represent means ±SD from 3 to 4 principal cells (3 ≤ *n* ≤ 4). Asterisks denote statistical significance in pairwise comparisons (ns = not significant; two-tailed, unpaired Student’s *t*-test).

**
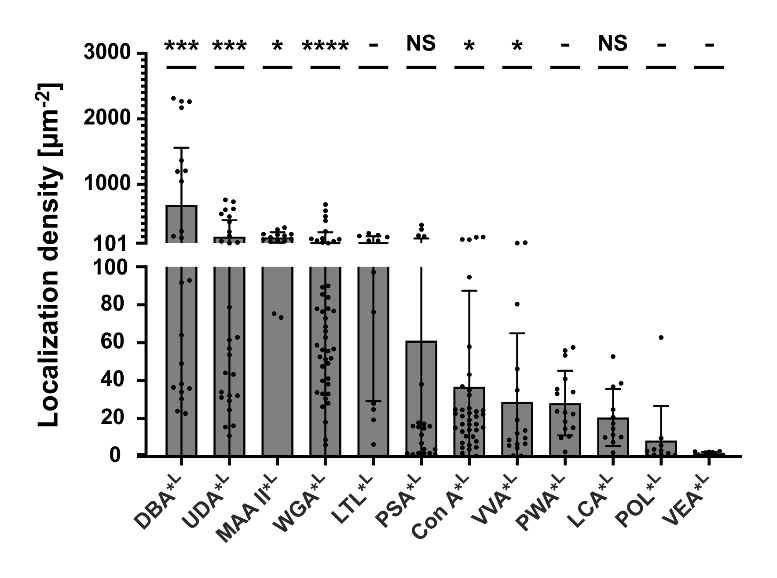
**

**Figure S9.** Signal density of lectins in the nucleus. Density of localizations within the nuclear area. Bar plots represent means ±SD from 3 to 8 independent experiments per lectin (3 ≤ *N* ≤ 8) and 3 to 8 measured areas per experiment (3 ≤ *n* ≤ 8). Asterisks denote statistical significance (****p ≤ 0.0001, ***p ≤ 0.001, *p ≤ 0.05, NS = not significant; unpaired one-tailed Mann-Whitney U test) versus nonspecific signal on glass surface (not shown for clarity). Dashes (-) indicate that no reference for nonspecific signal is available for the respective lectins due to the absence of regions lacking tissue in these experiments.

**
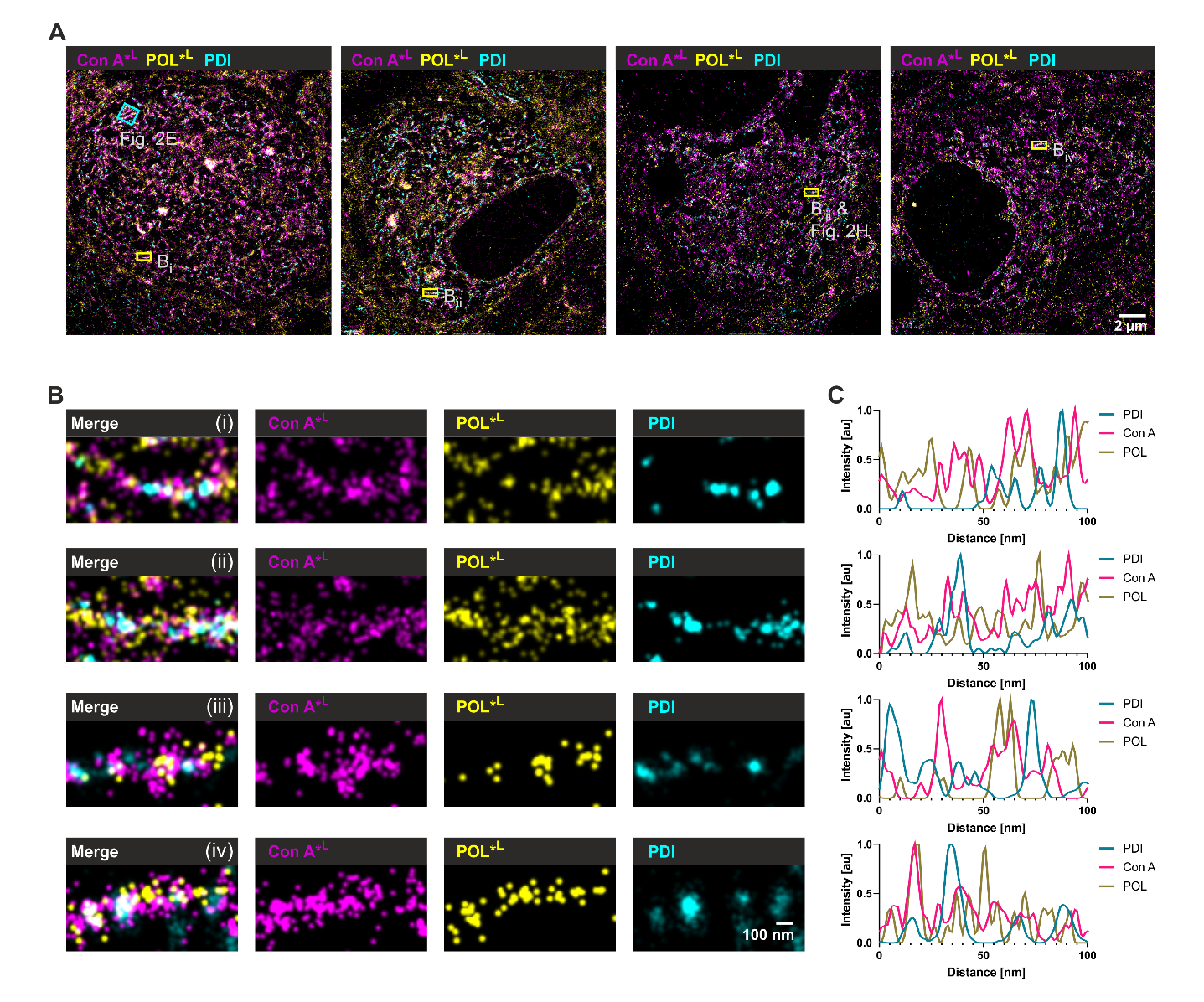
**

**Figure S10.** Reproducibility of lectins Con A and POL with PDI staining across cells and biological replicates. A) Exemplary *d*STORM overview images of Con A, POL, and PDI staining in neuronal tissue, acquired from two independent experiments with two images/cells per experiment (Exp. 3: panels 1 and 2; Exp. 7: panels 3 and 4; see Table S8). B) Magnified views of endoplasmic reticulum (ER) tubules from the corresponding images. C) Line profiles across selected tubules, with intensities normalized to 1.

**
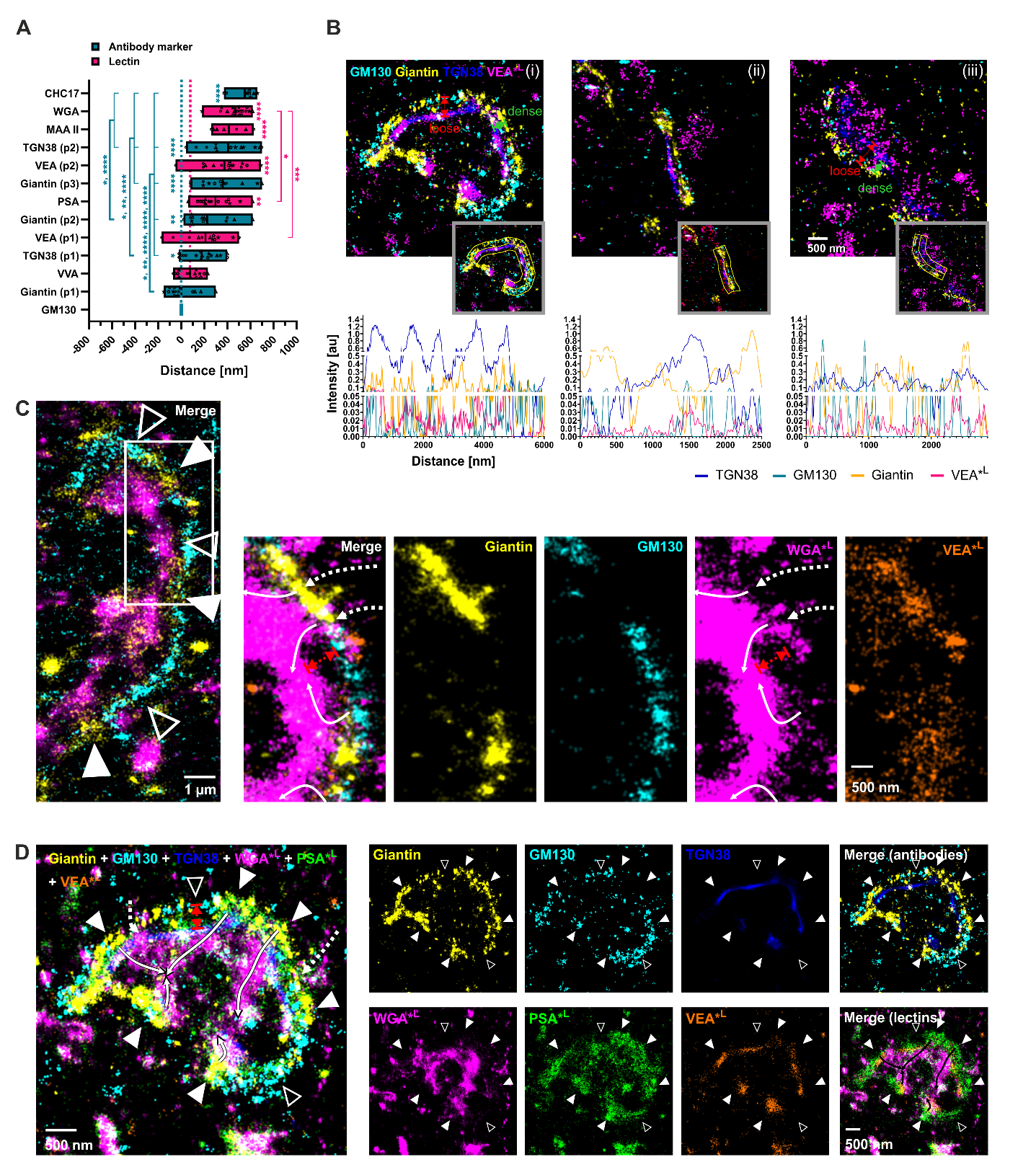
**

**Figure S11.** Lateral Golgi organization in brain tissue neurons. A) Bar graph showing the mean distance of the maxima of averaged line profiles relative to the *cis*-Golgi marker GM130 (gray line). Each data point corresponds to the maximum position averaged per Golgi apparatus; Golgi regions from 7 to 17 principal cells were analyzed per label (7 ≤ *n* ≤ 17); Data were collected across 2–5 independent experiments (2 ≤ *n* ≤ 5), which are distinguished by different symbols (circle, triangle, square, pentagon, or star). Note that some lectins and antibody markers showed multiple peaks (p1–3). Asterisks denote statistical significance from comparisons either among antibody markers (magenta) or lectins (cyan) (****p ≤ 0.0001, ***p ≤ 0.001, **p ≤ 0.01, *p ≤ 0.05, ns = not significant; one-way ANOVA with Tukey’s post hoc test). Asterisks on bars denote significance versus GM130 (cyan) or versus VVA (magenta). B-D) *d*STORM examples illustrating the lateral periodic distribution of Golgi antibody markers GM130, Giantin, and TGN38 and lectins. GM130 and Giantin signals are periodically alternating along the lateral Golgi ribbon axis (open and filled arrowheads in C and D). In many experiments, VEA signal positively correlates with Giantin. B) The top row shows example images, while the bottom row presents line profiles taken along the lateral Golgi axis (shown in insets of the top-row images). Often Golgi regions overarched by Giantin are more densely packed along the *cis*-to-*trans* axis (denoted by green double-headed arrows between GM130 and TGN38 markers in i and iii) compared to the looser packaging in GM130-rich regions with low or absent Giantin content (red double-headed arrows). D,E) Two examples illustrating how WGA-positive vesicles could traverse Giantin-overarched regions (dashed and solid white curved arrows), eventually merging with the TGN region. We propose that lectins such as VEA and WGA that appear enriched in Giantin-enclosed Golgi areas could represent non-compact Golgi regions.


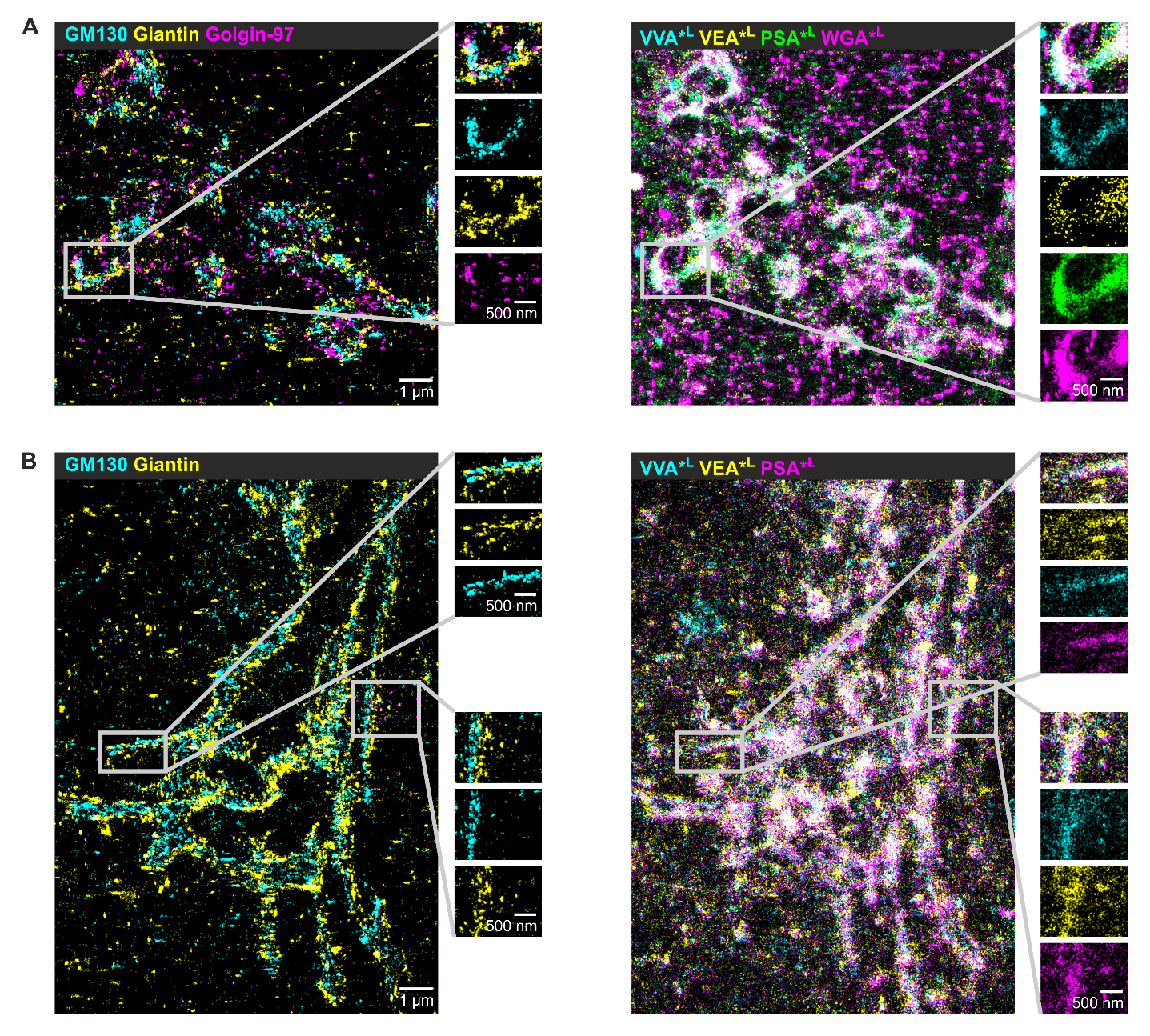


**Figure S12.** Golgi in U2OS and hippocampal neurons. Golgi apparatus ultrastructure visualized by conventional antibody markers and lectins in (A) human osteosarcoma cells (U2OS) and (B) hippocampal neurons.


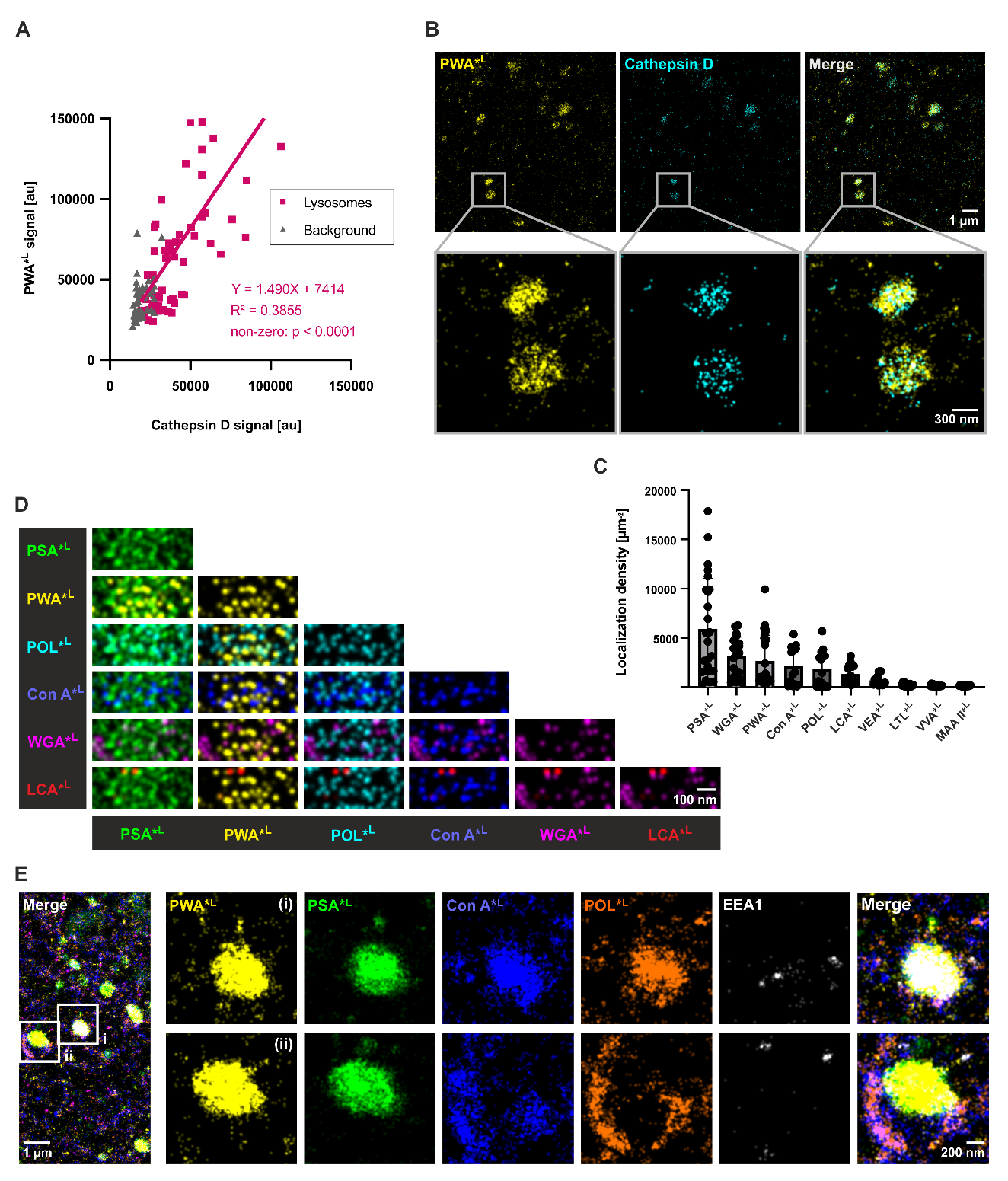


**Figure S13.** Pre-lysosomal compartments/lysosomes in brain tissue cell somata. A) PWA signal correlates with pre-lysosomal compartment (PLC) and lysosome marker Cathepsin D. Clusters were chosen based on areas enriched for PWA signal from wide-field images. Total number of selections *n* = 59 from 5 experiments (*N* = 5). B) Glyco-STORM image illustrating that PWA is specifically present in lysosomes identified by the Cathepsin D antibody. However, PWA and Cathepsin D nanodomains do not overlap. C) Density of localizations per lysosome quantified from *d*STORM images. Number of selections and individual experiments: PWA, PSA – *n* = 25, *N* = 5; Con A, POL, LCA, VEA, LTL, VVA, MAA II – *n* = 15, *N* = 3; WGA – *n* = 20, *N* = 4. D) Pairwise merging of lectin signals from an optical cross-section through a 3D image of the lysosome. E) Two examples of PWA-positive PLCs/lysosomes, showing distinct staining patterns of the mannose-binding lectins Con A and POL: (i) luminal signal within the organelle and (ii) surrounding signal that forms a clasp-like structure around the organelle.


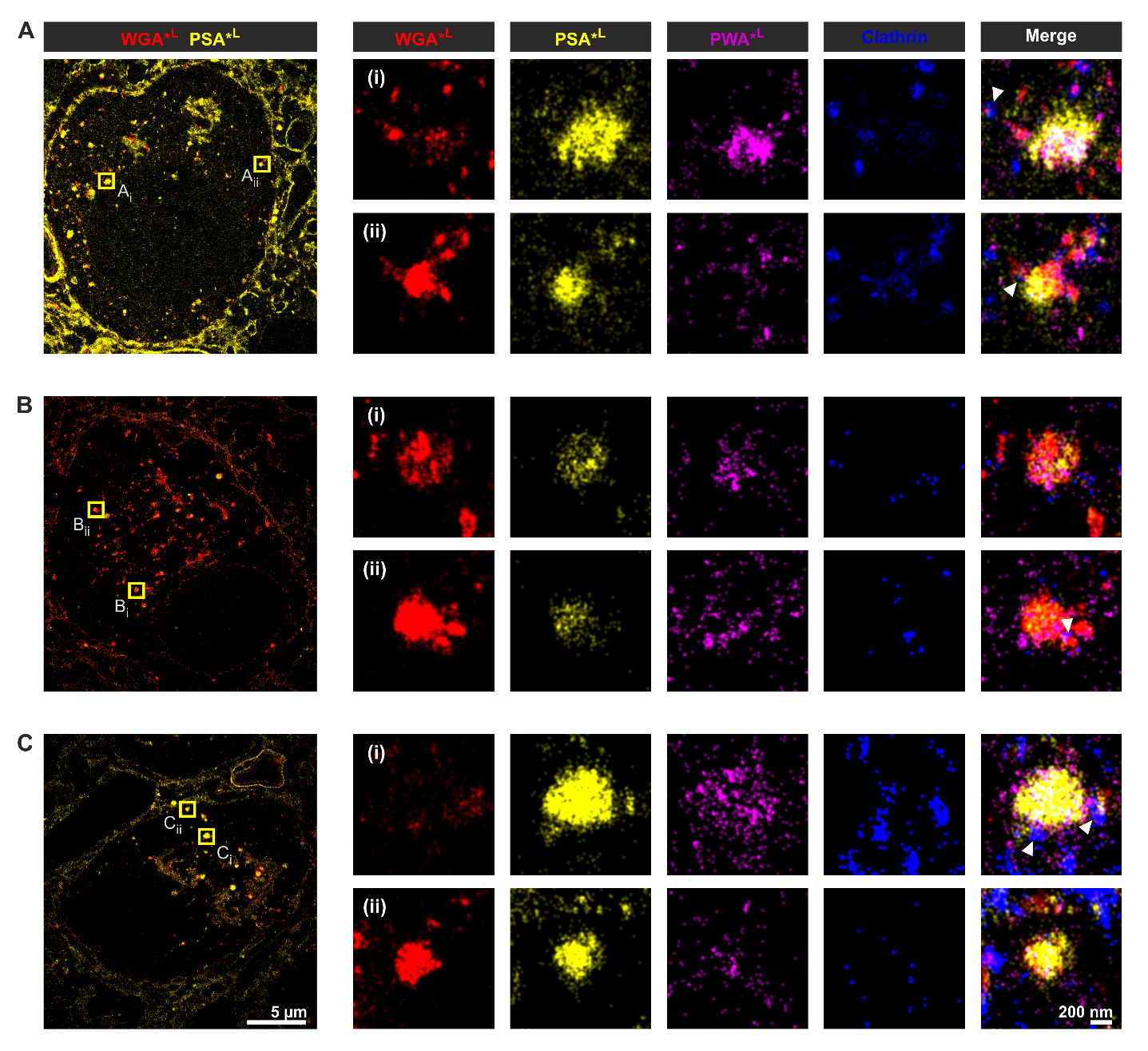


**Figure S14.** Round organelle staining patterns across biological replicates. A-C) Representative *d*STORM datasets of lectins WGA, PSA, and PWA and clathrin marker CHC17 acquired in three independent experiments (A, B, C). Left panels show overview images; yellow squares indicate regions magnified in (i) and (ii). Despite variability in overall signal intensities and organelle abundance across experiments, the principal spatial relationships are retained. Two classes are observed: (i) larger round organelles with higher PWA signal and WGA enriched at the periphery, corresponding to presumptive (pre-)lysosomes, and (ii) smaller organelles with stronger WGA signal. Clathrin decoration is exemplarily denoted by arrow heads.


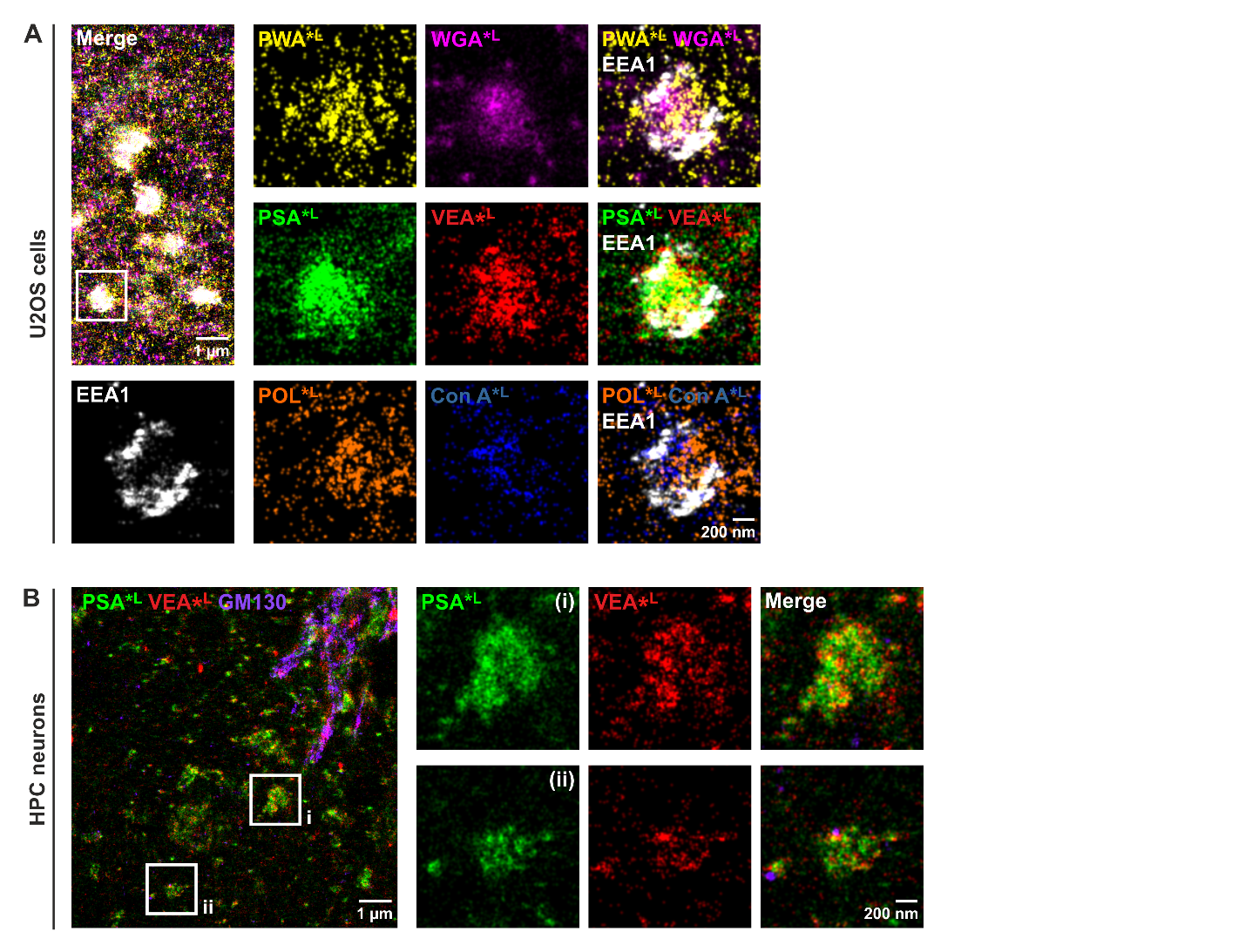


**Figure S15.** Glyco-STORM of pre-lysosomal compartments/lysosomes in U2OS and hippocampal cells. A) In U2OS cells, multiple lectins stained large spherical organelles, with most of these organelles being abundantly coated by the early endosome marker EEA1. B) In hippocampal (HPC) neurons, spherical organelles of different sizes, located in areas distinct from the main Golgi body indicated by GM130 staining, were positive for pre-lysosomal compartment/lysosome-binding lectins PSA and VEA.

**
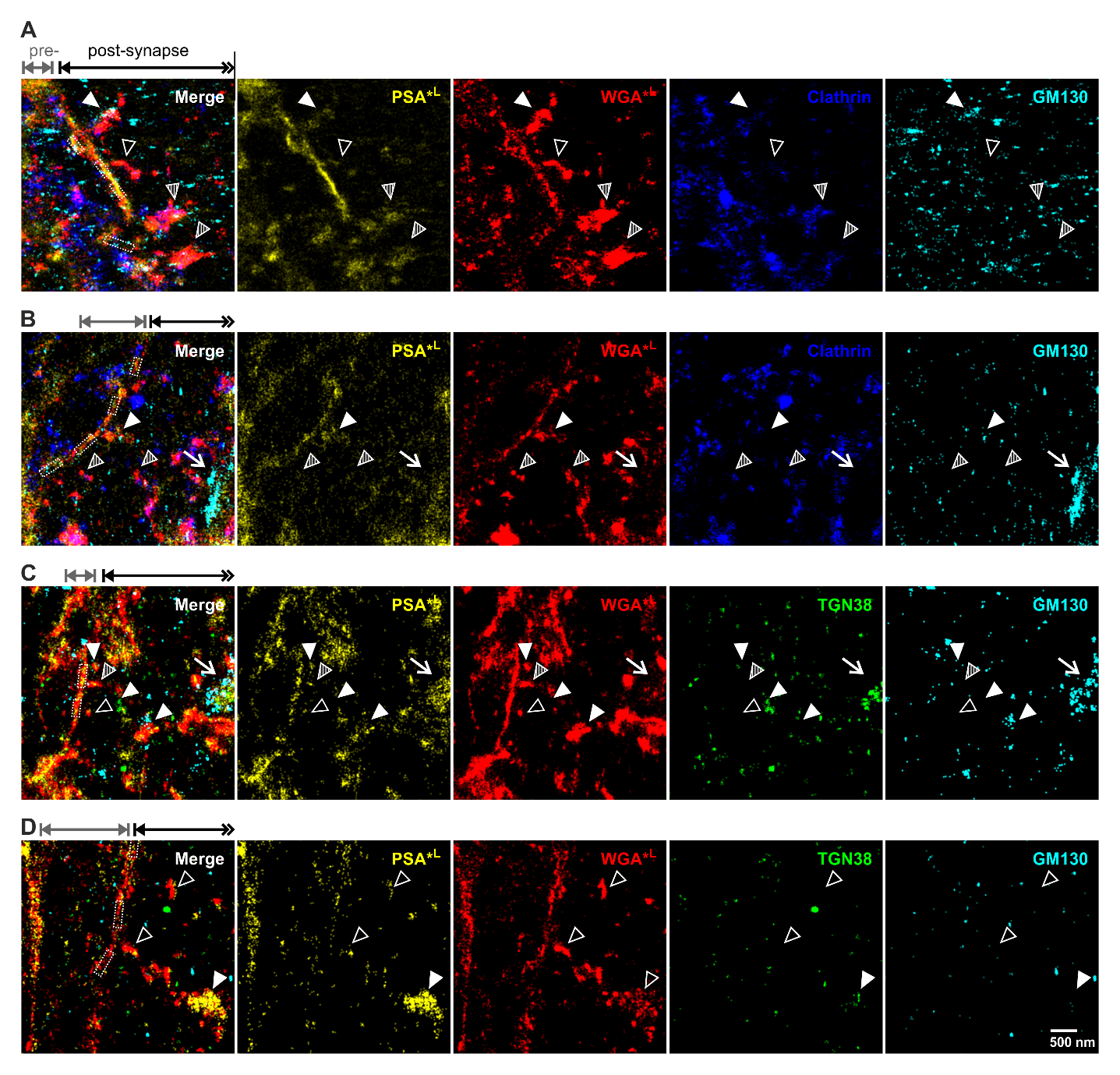
Figure S16.** Tubular glycan-rich perisynaptic structures in the postsynaptic principal cell of the giant glutamatergic synapse in brain tissue. A-D) Examples of postsynaptic glycans in proximity to synaptic contacts (dashed boxes; approximated based on Homer signal, not shown here). Some peri-synaptic glycan structures contain traces of Golgi markers GM130 and/or TGN38 (filled arrowheads), others do not coincide with these Golgi markers (open arrowheads). Many peri-synaptic glycan structures also contain clathrin traces (C,D). Arrows point to Golgi marker signal from the main Golgi apparatus whose parts are included in some images (B,C).

**
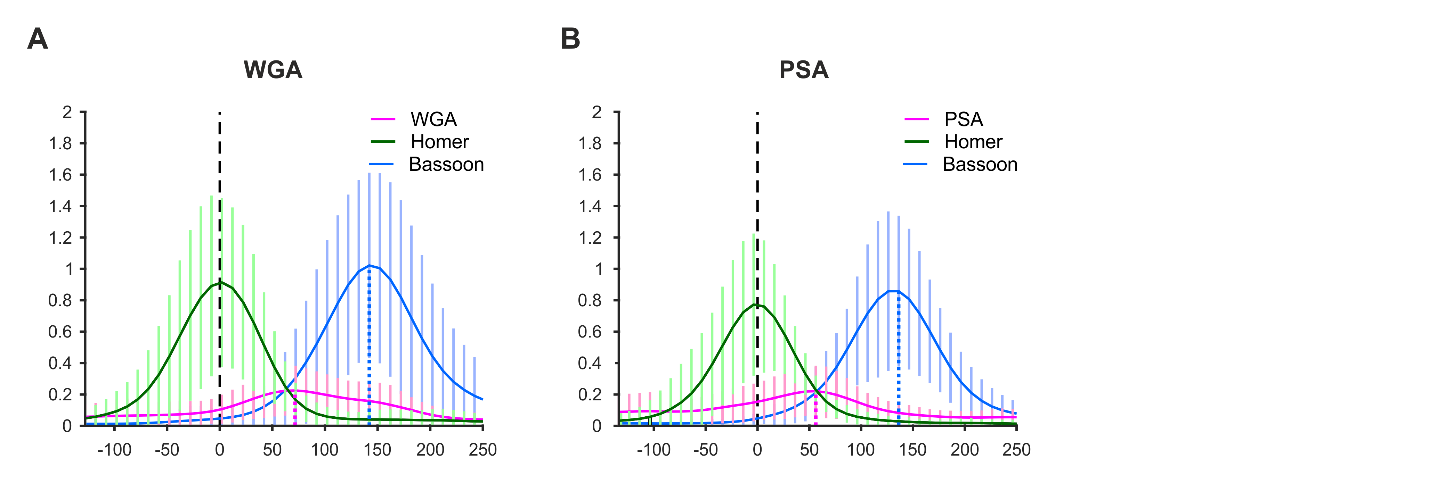
**

**Figure S17.** Distribution of glycans at the synaptic cleft of hippocampal neurons. Averaged line profiles demonstrating that both WGA (A) and PSA (B) peak in the center between the active zone and the postsynaptic density. Line profiles for Homer and Bassoon were averaged from *n* = 31 intensity profiles that are in turn averages for 3–10 synaptic interfaces per image acquired in *N* = 12 individual experiments (*η* = 110 synaptic interfaces in total); WGA: *η* = 91, *n* = 22, *N* = 8; PSA: *η* = 32, *n* = 19, *N* = 5. Vertical lines represent SD. The plotted peaks of the averaged profiles are displayed in the main Figure 4H.

**
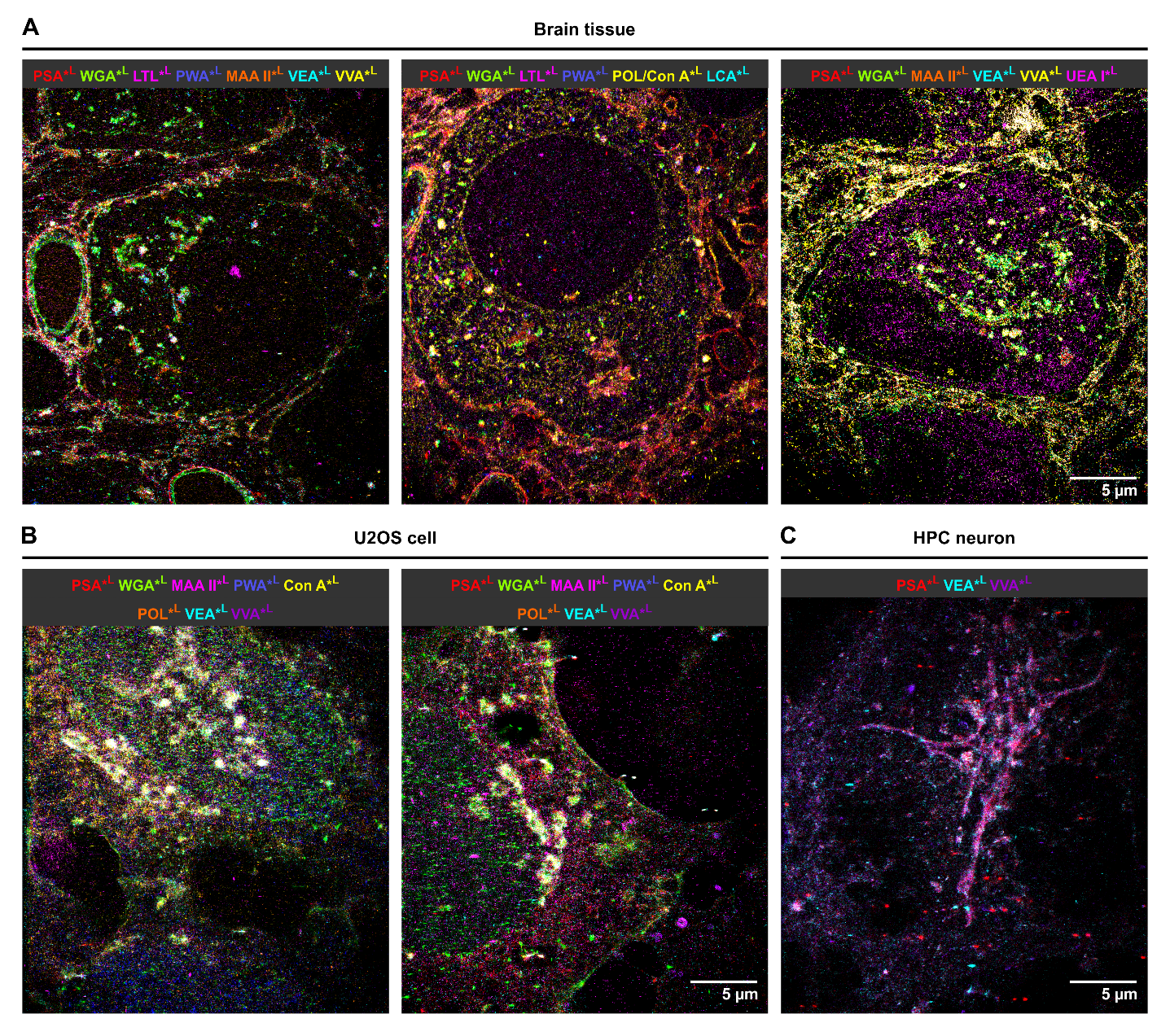
**

**Figure S18.** Glycosylation maps. A) Examples from three individual experiments conducted with different lectin subsets in neuronal tissue. B,C) Exemplary glycosylation landscapes of two U2OS cells (B) and a hippocampal (HPC) neuron (C).

**
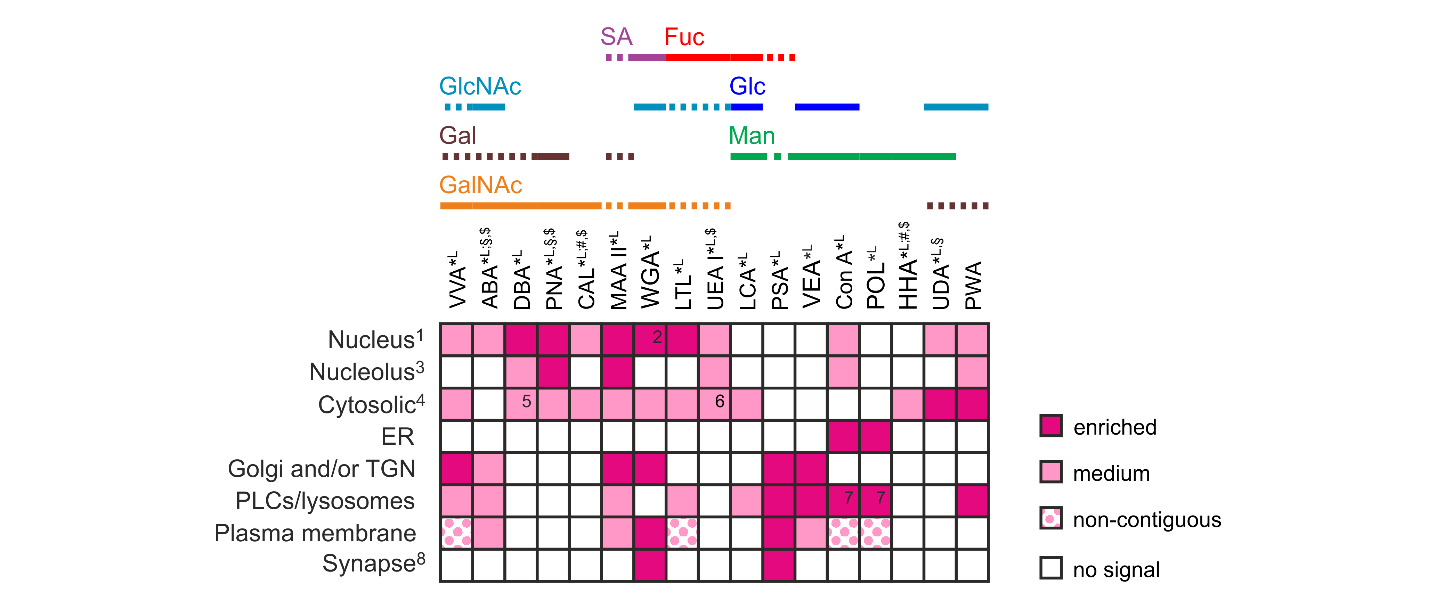
Figure S19.** Performance matrix for all screened lectins. Lectin performance matrix for 17 tested lectins, including those with low-intensity signals (^#^), variable performance (^$^), and those that were evaluated predominantly based on confocal screening (^§^). Note that a subset of lectins shown here is also highlighted in Figure 5B as the best-studied examples tested by Glyco-STORM. ^1^Nucleus staining refers to clustered or dispersed signal in the nucleoplasm if not stated otherwise. ^2^In addition to the clustered signal in the nucleoplasm, WGA stains the nuclear pores. ^3^Nucleolar signal was denoted as present if detected in a subset of cells. ^4^Cytosolic localization refers to clustered or dispersed signal patterns. ^5^DBA showed abundant cytosolic signal sparing distinct areas, including the Golgi apparatus. ^6^UEA I showed streaky signal in U2OS cells and hippocampal neurons and clustered round signal in the principal cell soma in brain tissue. ^7^For some pre-lysosomal compartments/lysosomes, Con A and POL showed signals within the lumen of the organelle, while in others their signals were located at the periphery. ^8^Synaptic localization applies only to brain tissue model. Solid lines highlight predominant sugar specificity; dashed lines denote sugar moieties that are primarily bound as part of a glycan motif. Abbreviations: ER, endoplasmic reticulum; Fuc, fucose; Gal, galactose; GalNAc, N-acetylgalactosamine; Glc, glucose; GlcNAc, N-acetylglucosamine; Man, mannose; PLCs, pre-lysosomal compartments; SA, sialic acid; TGN, *trans*-Golgi network.

# Supplementary References

1. Heinze KG, Costantino S, De Koninck P, Wiseman PW. Beyond Photobleaching, Laser Illumination Unbinds Fluorescent Proteins. J Phys Chem B; 2009;113(15):5225–33.

2. Klevanski M, Herrmannsdoerfer F, Sass S, Venkataramani V, Heilemann M, Kuner T. Automated highly multiplexed super-resolution imaging of protein nano-architecture in cells and tissues. Nat Commun; 2020;11(1):1552.
