# Supplementary material for: Nanoscale Mapping of the Subcellular Glycosylation Landscape
Source: Adv Sci (Weinh). 2025 Nov 10;13(11):e06731. doi: 10.1002/advs.202506731 (PMC12931256; doi:10.1002/advs.202506731)
Supplement: Supplementary file 3 — Supporting Tables [file ADVS-13-e06731-s003.docx]

**Supplementary Tables**

## Supplementary Table 1 | Properties of lectins used in this study

| **Lectin;  Organism** | **Size [kDa]; Subunits** | **Preferred binding motifs** |
| --- | --- | --- |
| ABA;   *Agaricus bisporus* | 60; 4^[1]^ | N-glycans: biantennary, β-GlcNAc terminated;^[2]^ N-glycans: biantennary, LacNAc terminated;^[2]^  O-glycans: core 2 (GlcNAcβ1-6(Galβ1-3)GalNAc)^[2]^ |
| CAL;   *Cicer arietinum* | 43; 2^[3]^ | N-glycans: complex carbohydrates;^[3]^  GalNAc^[4]^ |
| Con A;   *Canavalia ensiformis* | 104; 4^[5]^ | N-glycans: α-Man, terminal (Man_3_ to Man_9_);^[2]^ N-glycans: biantennary with most extensions (no terminus-proximal α-Fuc)^[2]^ |
| DBA;   *Dolichos biflorus* | 111; 4^[6]^ | Forssman antigen (GalNAcα1-3GalNAcβ1-3Galα1-4Galβ1-4Glc);^[2,7]^ GalNAcα1-3GalNAc;^[7]^  β-GalNAc;^[2]^ α-GalNAc;^[7,8]^  GalNAcα1-3(Fucα1-2)Gal^[7]^ |
| HHA;   *Hippeastrum hybrid* | 48; 4^[9]^ | N-glycans: Man, terminal: Manα1-6(Manα1-3)Man;^[2]^ Man_5_>Man_6_>Man_7_^[2]^ |
| LCA;   *Lens culinaris* | 48; 4^[9]^ | N-glycans: Fuc, core (Fucα1-6) ;^[2]^  Man, terminal (Manα1-2)^[2]^ |
| LTL;   *Lotus tetragonolobus* | 105; 4^[10]^ | Lewis^X^: Galβ1-4(Fucα1-3)GlcNAc;^[2]^ Fucα1-3;^[2]^ Lewis^y^: Fucα1-2Galβ1-4(Fucα1-3)GlcNAc^[2]^ |
| MAA II;  *Maackia amurensis* | 130; 4^[11]^ | O-Glycan: SA α2-3-linked to Galβ1-3GalNAc;^[2]^  3’ sulfated β-Gal^[2]^ |
| PNA;   *Arachis hypogaea* | 110; 4 | O-Glycan: Galβ1-3GalNAc, terminal^[2]^ |
| POL;   *Polygonatum odoratum* | 48; 4^[12]^ | N-glycans: Man_3_ (Manα1-3(Manα1-6)Man);^[12]^ Man_2_>Man_1_^[12]^ |
| PSA;   *Pisum sativum* | 50; 4^[13]^,^[14]^ | Fuc, core (Fucα1-6), tolerating SA, Gal, GlcNAc;^[2]^  Man^[15]^ |
| PWA;   *Phytolacca americana* | 32; 1^[16]^ | Chitin oligomers (GlcNAcβ1-4)_n_ (n ≥ 4), terminal^[2]^ poly-LacNAc^[17]^ |
| UDA;   *Urtica dioica* | 9; 1^[18]^ | Manα1-6, terminal Man_3_ to Man_9_, with chitobiose core;^[2]^ (poly-)LacNAc;^[2]^ Chitin oligomers (GlcNAcβ1-4)_n_^[2]^ |
| UEA I;   *Ulex europaeus* | 63; 2^[19]^ | Type 2 blood group H (Fucα1-2Galβ1-4GlcNAc);^[2]^  Fuc (Fucα1-2Galβ1-4Glc);^[2]^ Lewis^y^ (Fucα1-2Galβ1-4(Fucα1-3)GlcNAc)^[2]^ |
| VEA;   *Vicia ervilia* | 60; 4^[20]^ | α-Man;^[20]^  α-Glc^[20,21]^ |
| VVA A+B;   *Vicia villosa* | 136; 4 & 144; 4^[9]^ | GalNAcβ1-4GlcNAc;^[2]^ α/β-GalNAc, terminal;^[2]^ LacNAc, terminal, multiantennary^[2]^ |
| WGA;   *Triticum aestivum* | 36; 2^[22]^ | β-GlcNAc, terminal;^[2]^ α-GlcNAc, terminal;^[2]^  α/β-GalNAc, terminal;^[2]^ SA, terminal: e.g. α-Neu5Ac^[2]^ |

The first entry for each lectin indicates the predominant binding motif. Abbreviations: Fuc, fucose; Gal, galactose; GalNAc, N-acetylgalactosamine; Glc, glucose; GlcNAc, N-acetylglucosamine; LacNAc, N-acetyllactosamine; Man, mannose; Neu5Ac, N-acetylneuraminic acid; SA, sialic acid.

## Supplementary Table 2 | Lectin-fluorophore conjugates used in this study

| **Lectin** | **Conjugate** | **Used dilution** | **Recommended/ applied ions** | **Order №** | **Lot №** |
| --- | --- | --- | --- | --- | --- |
| ABA | AF647 | 1:20 | - | 21511446^a^ | L20061809ZH |
| CAL | AF647 | 1:20 | Ca^2+^, Mg^2+^ | 21511589^a^ | L20110609ZH |
| Con A | AF647 | 1:20 | Ca^2+^, Mn^2+^ | 21511462^a^ | L20042403ZH |
| Con A | CF680 | 1:200 | Ca^2+^, Mn^2+^ | 29020^b^ | 21C0720-1164044 |
| DBA | AF647 | 1:20 | Ca^2+^, Mg^2+^, Mn^2+^, Zn^2+^ | 21511470^a^ | L20110605ZH |
| HHA | AF647 | 1:5 | - | 21511503^a^ | L20090406ZH |
| LCA | AF647 | 1:20 | Ca^2+^, Mg^2+^ | 21511511^a^ | L20110606ZH |
| LTL | AF647 | 1:20 | Ca^2+^, Mn^2+^ | 21511594^a^ | L20110612ZH |
| MAA II | AF647 | 1:20 | - | 21511531^a^ | L20110607ZH |
| PNA | AF647 | 1:20 | Ca^2+^, Mg^2+^ | 21511454^a^ | L20110604ZH |
| POL | AF647 | 1:20 | Ca^2+^, Mn^2+^ | 21511595^a^ | L20110613ZH |
| PSA | AF647 | 1:100 | Ca^2+^, Mn^2+^ | 21511543^a^ | L20110611ZH |
| PWA | AF647 | 1:20 | - | 21511588^a^ | L20110608ZH |
| UDA | AF647 | 1:100 | Zn^2+^ | 21511581^a^ | L20052206ZH |
| UEA I | DL649 | 1:20 | Ca^2+^, Mn^2+^, Zn^2+^ | BL-21068^c^ | ZD1130 |
| VEA | AF647 | 1:20 | - | 21511591^a^ | L20110610ZH |
| VVA A+B | AF647 | 1:5 | Ca^2+^, Mn^2+^ | 21511590^a^ | L21080900CRCR |
| WGA | CF680 | 1:1000 | Ca^2+^ | 29029^b^ | 20W0527-1122158 |

Lectins were purchased as fluorophore conjugates from: ^a^BioWORLD, Dublin, OH, USA, ^b^Biotium, Fremont, CA, USA, or ^c^Vector Laboratories, Burlingame, CA, USA.

## Supplementary Table 3 | Primary antibodies and labels used in this study

| **Antibody/ Label** | **Sub-type** | **Host** | **Clonality, №** | **Used dilution** | **Provider** | **Order №** | **Lot №** |
| --- | --- | --- | --- | --- | --- | --- | --- |
| **Fluorophore-conjugated primary antibodies or other direct labels** | | | | | | | |
| CHC17- AF647 | IgG1 | ms | mono, X22 | 1:30 | Novusbio | NB300-613AF647 | VL315162-070121-AF647 |
|  |  |  |  |  |  |  |  |
| DAPI | toxin | - | - | 1:5000 of  50 mg/ml | Invitrogen | D1306 | - |
| Hoechst-JF646 | Bisbenz-imide | - | - | 100 pM | Luke Lavis | - | - |
| Phalloidin-AF647 | toxin | - | - | 1:80 | ThermoFisher | A22287 | 1750839 |
| Phalloidin-AF680 | toxin | - | - | 1:80 | ThermoFisher | A22286 | 1709895 |
| VGlut1-AF647 | sdAB | ll | mono, Nb9 | 1:200 | NanoTag | N1605 | 220602 |
| VGlut1-CF680 | sdAB | ll | mono, Nb9 | 1:200 | NanoTag | N1605 | 12190101 |
| **Unconjugated primary antibodies** | | | | | | | |
| Bassoon | IgG2aκ | ms | mono, SAP7F407 | 1:200 | Enzo Life Sciences | ADI-VAM-PS003 | 02012005 |
| Cathepsin D | IgG | rb | poly | 1:200 | Cell Signaling | 69854 | 1 |
| EEA1 | IgG | rb | poly | 1:200 | Abcam | ab2900 | - |
| GLT1 | serum | gp | poly | 1:400 | Millipore | AB1783 | 3526283 |
| Fibrillarin | IgG | rb | poly | 1:200 | Abcam | ab5821 | GR293483-2 |
| Giantin | serum | gp | poly | 1:200 | SySy | 263004 | 263004/1 |
| GM130 | IgG1 | ms | mono, 35 | 1:200 | BD Biosciences | 610822 | 1145724 |
| Golgin97 | IgG | rb | poly | 1:70 | ThermoFisher | PA5-83719 | - |
| Homer1b/c | IgG | rb | poly | 1:400 | SySy | 160023 | 160023/2-11 |
| Lamin a/c | IgY | ch | poly | 1:200 | Biosensis | C1698-100 | C-1698-300-201307-SH |
| LAMP1 | IgG1 | ms | mono, H4A3 | 1:200 | Abcam | ab25630 | GR3395210-1 |
| LAMP1 | IgG | rb | poly | 1:200 | Abcam | ab24170 | GR3395210-1 |
| LAMP2A | IgG | gp | poly | 1:400 | SySy | 437005 | - |
| LAMP3 | IgG | gp | poly | 1:200 | SySy | 391005 | - |
| LAMP5 | IgG | gp | poly | 1:200 | SySy | 412005 | - |
| MAP2 | IgY | ch | poly | 1:500 | SySy | 188006 | 188006/1 |
| PDI | IgG1 | ms | mono, 1D3 | 1:400 | Enzo Life Sciences | ADI-SPA-891 | 03022019 |
| PEX14 | IgG | rb | poly | 1:200 | Novusbio | NPB2-33455 | A119064 |
| Piccolo | IgG | rb | poly | 1:200 | SySy | 142113 | 142113/1-3 |
| Rab3a | IgG1 | ms | mono, 42.2 | 1:200 | SySy | 107111 | 107111/1-20 |
| Rab5 | IgG | rb | poly | 1:200 | Abcam | ab18211 | 793878 |
| Rab7 | IgG | rb | mono, n.a. | 1:200 | Abcam | ab137029 | GR155792-64 |
| S100B | IgY | ch | poly | 1:400 | SySy | 287006 | 287006/1-4 |
| SCAMP1 | IgG | rb | poly | 1:200 | SySy | 121003 | - |
| Synapto-physin | IgG | rb | poly | 1:200 | SySy | 101002 | 101002/26 or 1-41 |
| Syntaxin6 | serum | rb | poly | 1:100 | SySy | 110062 | - |
| SV2 a/b/c | IgG1 | ms | mono | 1:200 | DSHB | AB2315387 | ABC214744 |
| TGN38 | IgG1 | ms | mono, Clone 2 | 1:100-1:200 | BD Biosciences | 610898 | 8274719 |
| TGN38 | serum | rb | poly | 1:100 | Novus Biologicals | NB1-03495SS | RB0705-060908-WS |
| VAMP1 | serum | rb | poly | 1:500 | SySy | 104002 | 104002/1-16 |
| VAMP2 | IgG | rb | mono | 1:500 | SySy | 104008 | 104002/1-5 |
| VAMP4 | serum | rb | poly | 1:100 | SySy | 136002 | - |
| VAMP7 | IgG | ms | mono, 158.2 | 1:100 | SySy | 232011 | 232011/1-8 |
| VGlut1 | serum | gp | poly | 1:100 | Millipore | AB5905 | 3836436 |
| VGlut2 | IgY | ch | poly | 1:200 | SySy | 135416 | 135416/1-6 |

Abbreviations: AF647/680, Alexa Fluor 647/680; ch, chicken; gp, guinea pig; JF646, Janelia Fluor 646; ll, llama; mono, monoclonal; ms, mouse; n.a., not available; poly, polyclonal; rb, rabbit; sdAB, single domain antibody (nanobody); SySy, Synaptic Systems.

## Supplementary Table 4 | Secondary antibodies used in this study

| **Target/ Conjugate** | **Sub-type** | **Application** | **Host** | **Used dilution** | **Provider** | **Order №** | **Lot №** |
| --- | --- | --- | --- | --- | --- | --- | --- |
| anti-ms IgG/ AF488 | F(ab')_2_ | WF | gt | 1:400 | Invitrogen | A11017 | 262519 |
| anti-ms IgG/ AF647 | IgG (H+L) | WF | gt | 1:400 | Invitrogen | A21235 | 2369184 |
| anti-ms IgG/ AF647 | F(ab')_2_ | *d*STORM | dn | 1:400 | Abcam | ab181292 | GR3310174-2 |
| anti-ms IgG/ CF680 | IgG (H+L) | *d*STORM | gt | 1:400 | Sigma | SAB4600199 | 18C0130 |
|  |  |  |  |  |  |  |  |
| anti-rb IgG/ AF488 | IgG (H+L) | WF | gt | 1:400 | Invitrogen | A11008 | 2420731 |
| anti-rb IgG/ AF568 | IgG (H+L) | WF | gt | 1:400 | Invitrogen | A11011 | 2500544 |
| anti-rb IgG/ AF647 | IgG (H+L) | WF | gt | 1:400 | Invitrogen | A21245 | 264997 |
| anti-rb IgG/ AF647 | F(ab')_2_ | *d*STORM | dn | 1:400 | Abcam | ab181347 | GR3361186-1 |
| anti-rb IgG/ CF680 | F(ab')_2_ | *d*STORM | gt | 1:400 | Sigma | SAB4600362 | 16C0721 |
| anti-gp IgG/ AF488 | IgG (H+L) | WF | gt | 1:400 | Invitrogen | A11073 | 1990462 |
| anti-gp IgG/ CF680 | IgG (H+L) | *d*STORM | gt | 1:400 | Biotium | 20499 | 21C0104 |
|  |  |  |  |  |  |  |  |
| anti-ch IgY/ AF488 | IgG (H+L) | WF | gt | 1:400 | Invitrogen | A11039 | 2566343 |
| anti-ch IgY/ AF647 | IgG (H+L) | *d*STORM | gt | 1:400 | Invitrogen | A21449 | 1806124 |
|  |  |  |  |  |  |  |  |
|  |  |  |  |  |  |  |  |

All secondary antibodies used in this study were polyclonal. AF488/568/647, Alexa Fluor 488/568/647; ch, chicken; dn, donkey; gp, guinea pig; gt, goat; ms, mouse; rb, rabbit; WF, wide-field.

## Supplementary Table 5 | Composition of lectin elution buffers

| **Eluted lectin** | **Competing sugars** | **Order №** |
| --- | --- | --- |
| ABA | 0.2 M Gal-GalNAc^a^ | 21511278-1 |
| CAL | 0.2 M Bovine fetuin^a^ | 21511283-1 |
| Con A, HHA, PSA, LCA | 0.2 M α-Methylmannoside^a^ | 21511337-1 |
| DBA | 0.2 M GalNAc^a^ | 21511031-1 |
| MAA II | 0.2 M Lactose^a^ | 21511261-1 |
| PNA | 0.2 M Galactose^a^ | 21511342-1 |
| POL | 0.1 M Mannose^b^ | 20120110-1 |
| PWA, WGA | 0.2 M Chitin hydrolysate^a^ | 21511023-3 |
| UDA | 0.2 M Chitobiose^a^ | 21511315-1 |
| UEA I, LTL | 0.2 M L-Fucose^a^ | 21511316-1 |
| VEA | 0.2 M Glucose^a^ | 21511318-1 |
| VVA | 0.1 M GalNAc^b^ | 21510884-1 |
| WGA | 0.1 M GlcNAc^b^ | 21510885-1 |

Competing sugars were purchased in the following buffers:
^a^ 10 mM Tris with 0.1 M or 1 M NaCl and 1 mM or 2 mM EDTA
^b^ 1 M NaCl and 2 mM EDTA

## Supplementary Table 6 | Areas segmented for region-specific elution analysis

| **Eluted lectin** | **Segmented area** |
| --- | --- |
| Con A | Endoplasmic reticulum, round organelles |
| DBA | Cytosol |
| LCA | Round organelles |
| MAA II | Golgi apparatus, plasma membrane |
| POL | Endoplasmic reticulum, round organelles |
| PSA | Golgi |
| PWA | Round organelles (lysosomes) |
| UEA I | Round organelles |
| VEA | Golgi apparatus |
| VVA | Golgi apparatus |
| WGA | Endoplasmic reticulum, round organelles, nuclear pores |

## Supplementary Table 7 | Number of experiments and confocal or wide-field images acquired during initial lectin screening and elution

| **Lectin** | **Brain tissue** | **Hippocampal neurons** | **U2OS cells** |
| --- | --- | --- | --- |
| ABA | *N* = 7 | *N* = 2 | *N* = 2 |
| CAL | *N* = 2 | *N* = 2 | *N* = 1 |
| Con A | *N* = 22 | *N* = 7 | *N* = 4 |
| DBA | *N* = 12 | *N* = 4 | *N* = 6 |
| HHA | *N* = 4 | *N* = 1 | *N* = 1 |
| LCA | *N* = 10 | *N* = 3 | *N* = 5 |
| LTL | *N* = 7 | *N* = 3 | *N* = 5 |
| MAA II | *N* = 21 | *N* = 3 | *N* = 9 |
| PNA | *N* = 5 | *N* = 2 | *N* = 7 |
| POL | *N* = 11 | *N* = 8 | *N* = 3 |
| PSA | *N* = 16 | *N* = 13 | *N* = 11 |
| PWA | *N* = 11 | *N* = 5 | *N* = 9 |
| UDA | *N* = 6 | *N* = 4 | *N* = 1 |
| UEA I | *N* = 7 | *N* = 4 | *N* = 4 |
| VEA | *N* = 17 | *N* = 12 | *N* = 11 |
| VVA A+B | *N* = 21 | *N* = 12 | *N* = 11 |
| WGA | *N* > 30 | *N* = 15 | *N* = 11 |

*N* is the total number of experiments with 5 – 7 cells each.

## Supplementary Table 8 | Workflows for Glyco-STORM experiments in brain tissue

| **Round** | **Label in channel 1** | **Label in channel 2** | **Signal removal  (post-imaging)** |
| --- | --- | --- | --- |
| ***Experiment 1*** *used in Fig. 2A-C, Fig. S9 (nucleus)* | | | |
| A | MAA II-AF647 | - | Bleaching |
| C | (n.r.) | WGA-CF680 | Bleaching |
| D | Con A-AF647 | Fibrillarin::rb-CF680 | Bleaching |
| E | UDA-AF647 | Bassoon::ms-CF680 | Bleaching |
| F | (n.r.) | VGlut1::gp-CF680 | Elution, Bleaching |
| H | MAA II-AF647 | (n.r.) | Elution, Bleaching |
| I | Hoechst-JF646 | Synaptophysin::rb-CF680 | - |
| J | Hoechst-JF646 | - | - |
| ***Experiment 2*** *used in Fig. 2C, Fig. S9 (nucleus)* | | | |
| A | DBA | Fibrillarin::rb-CF680 | Bleaching |
| B | Phalloidin-AF647 | WGA-CF680 | Bleaching |
| C | UDA-AF647 | SV2::ms-CF680 | Bleaching |
| D | Con A-AF647 | (n.r.) | Elution, Bleaching |
| E | (n.r.) | Bassoon::ms-CF680 | - |
| ***Experiment 3*** *used in Fig. 2C, Fig. S9 (nucleus); Fig. 2F-J (ER); Fig. 3C, Fig. S11B (Golgi); Fig. S13C (PLC/lysosome)* | | | |
| A | VVA-AF647 | - | Bleaching |
| B | VEA-AF647 | (n.r.) | Bleaching |
| C | PSA-AF647 | GM130::ms-CF680 | Bleaching |
| D | CHC17-AF647 | Giantin::gp-CF680 | Elution, Bleaching |
| E | PWA-AF647 | TGN38::ms-CF680 | Elution, Bleaching |
| F | MAA II-AF647 | (n.r.) | Bleaching |
| G | Con A-AF647 | WGA-CF680 | Elution, Bleaching |
| H | POL-AF647 | PEX14::rb-CF680 | Elution, Bleaching |
| I | PDI::ms-AF647 | - | - |
| ***Experiment 4*** *used in Fig. 2C, Fig. S9 (nucleus)* | | | |
| A | VVA-AF647 | Bassoon::ms-CF680 | Bleaching |
| B | GM130::ms-AF647 | Golgin97::rb-CF680 | Elution, Bleaching |
| C | (n.r.) | WGA-CF680 | Elution, Bleaching |
| D | Con A-AF647 | Synaptophysin::rb-CF680 | - |
| ***Experiment 5*** *used in Fig. 2B,C, Fig. S9 (nucleus); Fig. 2F,G,J (ER); Fig. 3B,C, Fig. S11D (Golgi); Fig. 3D-G, Fig. S13C,D (PLC/lysosome); Fig. 5A, Fig. S18A (glycosylation map); Fig. S6C,D (precision analysis); Fig. S16A,B (perisynaptic structures)* | | | |
| A | LCA-AF647 | - | Bleaching |
| B | Phalloidin-AF647 | LAMP1::rb-CF680 | Bleaching |
| C | PWA-AF647 | LAMP3::gp-CF680 | Bleaching |
| D | CHC17-AF647 | WGA-CF680 | Elution, Bleaching |
| E | VGlut2::ch-AF647 | Bassoon::ms-CF680 | Elution, Bleaching |
| F | LTL-AF647 | EEA1::rb-CF680 | Elution, Bleaching |
| G | VAMP7::ms-AF647 | Homer1b/c::rb-CF680 | Elution, Bleaching |
| H | POL-AF647 | Rab5::rb-CF680 | Elution, Bleaching |
| I | PSA-AF647 | GM130::ms-CF680 | Elution, Bleaching |
| J | Con A-AF647 | Rab7::rb-CF680 | - |

| ***Experiment 6*** *used in Fig. 2B,C, Fig. S9 (nucleus); Fig. 2F,G (ER)* | | | |
| --- | --- | --- | --- |
| A | Con A-AF647 | - | Bleaching |
| B | (n.r.) | Bassoon::ms-CF680 | Bleaching |
| C | Rab7::rb-AF647 | VGlut1::gp-CF680 | Elution, Bleaching |
| D | LAMP1::rb-AF647 | (n.r.) | Elution, Bleaching |
| E | PDI::ms-AF647 | WGA-CF680 | - |
| ***Experiment 7*** *used in Fig. 2B,C, Fig. S9 (nucleus); Fig. 2E-G,J (ER); Fig. S13C,E (PLC/lysosome)* | | | |
| A | PSA-AF647 | LAMP1::ms-CF680 | Bleaching |
| B | LCA-AF647 | LAMP5::gp-CF680 | Bleaching |
| C | PWA-AF647 | SCAMP1::rb-CF680 | Bleaching |
| E | LTL-AF647 | LAMP2A::gp-CF680 | Elution, Bleaching |
| F | Con A-AF647 | EEA1::rb-CF680 | Elution, Bleaching |
| G | POL-AF647 | PDI::ms-CF680 | - |
| ***Experiment 8*** *used in Fig. 2B,C, Fig. S9 (nucleus); Fig. 3C, Fig. S11B (Golgi); Fig. S18A* *(glycosylation map); Fig. S13C (PLC/lysosome); Fig. S16C,D (perisynaptic structures)* | | | |
| A | VEA-AF647 | TGN38::ms-CF680 | Bleaching |
| B | VVA-AF647 | WGA-CF680 | Bleaching |
| C | PSA-AF647 | Giantin::gp-CF680 | Bleaching |
| D | PWA-AF647 | Homer1b/c::rb-CF680 | Elution, Bleaching |
| F | SV2::ms-AF647 | (n.r.) | Elution, Bleaching |
| H | MAA II-AF647 | GM130::ms-CF680 | Elution, Bleaching |
| K | LTL-AF647 | (n.r.) | - |
| ***Experiment 9*** *used in Fig. 2C, Fig. S9 (nucleus); Fig. 3C, Fig. S11B,C (Golgi); Fig. S18A (glycosylation map); Fig. S13C (PLC/lysosome)* | | | |
| A | VVA-AF647 | - | Bleaching |
| B | VEA-AF647 | (n.r.) | Bleaching |
| C | PSA-AF647 | WGA-CF680 | Bleaching |
| D | MAA II-AF647 | Giantin::gp-CF680 | Elution, Bleaching |
| E | Golgin97::rb-AF647 | TGN38::ms-CF680 | Elution, Bleaching |
| F | CHC17-AF647 | VAMP4::rb-CF680 | Elution, Bleaching |
| G | UEA I-DL649 | GM130::ms-CF680 | - |
| ***Experiment 10***  *used in Fig. 2C, Fig. S9 (nucleus); Fig. S13C (PLC/lysosome)* | | | |
| A | PSA-AF647 | - | Bleaching |
| B | Bassoon::ms-AF647 | (n.r.) | Bleaching |
| C | LCA-AF647 | WGA-CF680 | Elution, Bleaching |
| D | GM130::ms-AF647 | LAMP1::rb-CF680 | Elution, Bleaching |
| E | Synaptophysin::rb-AF647 | GLT1::gp-CF680 | - |
| ***Experiment 11***  *used in Fig. 2C, Fig. S9 (nucleus)* | | | |
| A | ABA-AF647 | - | Bleaching |
| B | Bassoon::ms-AF647 | Homer1b/c::rb-CF680 | Bleaching |
| C | Synaptophysin::rb-AF647 | GLT1::gp-CF680 | Bleaching |
| D | S100B::ch-AF647 | WGA-CF680 | Elution, Bleaching |
| E | UDA-AF647 | Rab5::rb-CF680 | - |

| ***Experiment 12*** *used in Fig. 2C, Fig. S9 (nucleus); Fig. S6C,D (precision analysis)* | | | |
| --- | --- | --- | --- |
| A | - | Bassoon::ms-CF680  Piccolo::gp-CF680 (cumulative) | Bleaching |
| B | UDA-AF647 | - | Bleaching |
| C | (n.r.) | WGA-CF680 | - |
| ***Experiment 13*** *used in Fig. 2B,C, Fig. S9 (nucleus)* | | | |
| A | UDA-AF647 | - | Bleaching |
| B | MAP2::ch-AF647 | Bassoon::ms-CF680 | Bleaching |
| C | - | WGA-CF680 | Bleaching |
| D | Phalloidin-AF647 | Rab5::rb-CF680 | - |
| ***Experiment 14***  *used in Fig. 2B,C, Fig. S9 (nucleus); Fig. 2D,F,G (ER); Fig. S6C,D (precision analysis)* | | | |
| A | Con A-AF647 | - | Bleaching |
| B | - | Piccolo::gp-CF680 | Bleaching |
| C | PDI::ms-AF647 | WGA-CF680 | Bleaching |
| D | Synaptophysin::rb-AF647 | - | - |
| ***Experiment 15*** *used in Fig. 2C, Fig. S9 (nucleus)* | | | |
| A | UDA-AF647 | WGA-CF680 | - |
| ***Experiment 16*** | | | |
| A | - | WGA-CF680 | - |
| ***Experiment 17*** | | | |
| A | PWA-AF647 | - | Bleaching |
| B | PSA-AF647 | WGA-CF680 | - |
| ***Experiment 18*** *used in Fig. 2C, Fig. S9 (nucleus)* | | | |
| A | DBA-AF647 | - | Bleaching |
| B | Synaptophysin::rb-AF647 | PDI::ms-CF680 | Bleaching |
| C | MAP2::ch-AF647 | Phalloidin-AF680 | - |
| ***Experiment 19*** *used in Fig. S6C,D (precision analysis)* | | | |
| A | VVA-AF647 | - | Bleaching |
| B | Bassoon::ms-AF647 | (n.r.) | Bleaching |
| C | VGlut2::ch-AF647 | WGA-CF680 | Bleaching |
| D | VEA-AF647 | Giantin::gp-CF680 | Elution, Bleaching |
| E | GM130::ms-AF647 | Homer1b/c::rb-CF680 | Elution, Bleaching |
| F | (n.r.) | LAMP3::gp-CF680 | Elution, Bleaching |
| G | VAMP7::ms-AF647 | TGN38::rb-CF680 | Elution, Bleaching |
| H | MAA II-AF647 | Golgin97::rb-CF680 | - |
| ***Experiment 20*** *used in Fig. 4A,C (synapse)* | | | |
| A | VGlut1::gp-AF647 | Bassoon::ms-CF680 Piccolo::rb-CF680 (cumulative) | Bleaching |
| B | VGlut2::ch-AF647 | Homer1b/c::rb-CF680 | Elution, Bleaching |
| C | CHC17-AF647 | WGA-CF680 | Elution, Bleaching |
| D | Synaptophysin::rb-AF647 | (n.r.) | Elution, Bleaching |
| E | VAMP2::rb-AF647 | SV2::ms-CF680 | Elution, Bleaching |
| F | VAMP1::rb-AF647 | Rab3a::ms-CF680 | Elution, Bleaching |
| G | PSA-AF647 | (n.r.) | - |

| ***Experiment 21*** *(synapse)* | | | |
| --- | --- | --- | --- |
| A | VGlut1::gp-AF647 | Bassoon::ms-CF680 Piccolo::rb-CF680 (cumulative) | Bleaching |
| B | PSA-AF647 | WGA-CF680 | Elution, Bleaching |
| C | VGlut2::ch-AF647 | Homer1b/c::rb-CF680 | Elution, Bleaching |
| D | CHC17-AF647 | VGlut-CF680 | Elution, Bleaching |
| E | Synaptophysin::rb-AF647 | (n.r.) | Elution, Bleaching |
| F | VAMP2::rb-AF647 | SV2::ms-CF680 | Elution, Bleaching |
| ***Experiment 22*** *used in Fig. 2B,C, Fig. S9 (nucleus)* | | | |
| A | Bassoon::ms-AF647 | Piccolo::rb-CF680 | Elution, Bleaching |
| B | (n.r.) | Homer1b/c::rb-CF680 | Elution, Bleaching |
| C | Synaptophysin::rb-AF647 | WGA-CF680 | Elution, Bleaching |
| D | DBA-AF647 | (n.r.) | - |
| ***Experiment 23***  *used in Fig. S6C,D (precision analysis)* | | | |
| A | Piccolo::rb-AF647 | Bassoon::ms-CF680 | Elution, Bleaching |
| B | (n.r.) | Homer1b/c::rb-CF680 | Bleaching |
| C | VGlut-AF647 (NB) | VGlut-CF680 (NB) | Elution, Bleaching |
| D | Synaptophysin::rb-AF647 | WGA-CF680 | - |
| ***Experiment 24***  *used in Fig. S13A,B (PLC/lysosome)* | | | |
| A | PWA-AF647 | Cathepsin D::rb-CF680 | - |

In label names, a dash ‘-‘ symbolizes direct conjugation and a double colon ‘::’ symbolizes binding by a secondary antibody or nanobody. Abbreviations: AF647/680, Alexa Fluor 647/680; ch, chicken; gp, guinea pig; JF646, Janelia Fluor 646; ms, mouse; NB, nanobody; n.r., not relevant (if label did not work or has no relevance for this study); rb, rabbit.

## Supplementary Table 9 | Workflows for Glyco-STORM experiments in U2OS cells

| **Round** | **Label in channel 1** | **Label in channel 2** | **Signal removal  (post-imaging)** |
| --- | --- | --- | --- |
| ***Experiment 26;*** *used in Fig. S15A, Fig. S18B* | | | |
| A | VVA-AF647 | Golgin97::rb-CF680 | Bleaching |
| B | VEA-AF647 | GM130::ms-CF680 | Bleaching |
| C | PSA-AF647 | Giantin::gp-CF680 | Bleaching |
| D | CHC17-AF647 | WGA-CF680 | Elution, Bleaching |
| E | PWA-AF647 | TGN38::ms-CF680 | Elution, Bleaching |
| F | LAMP1::rb-AF647 | PDI::ms-CF680 | Elution, Bleaching |
| G | Con A-AF647 | EEA1::rb-CF680 | Elution, Bleaching |
| H | MAA II-AF647 | LAMP3::gp-CF680 | Elution, Bleaching |
| I | POL-AF647 | Rab7::rb-CF680 | - |
| ***Experiment 27;*** *used in Fig. S12A* | | | |
| A | VEA-AF647 | TGN38::ms-CF680 | Bleaching |
| B | VVA-AF647 | Golgin97::rb-CF680 | Bleaching |
| C | PSA-AF647 | GM130::ms-CF680 | Bleaching |
| D | CHC17-AF647 | Giantin::gp-CF680 | Bleaching |
| E | PWA-AF647 | WGA-CF680 | - |
| ***Experiment 28*** | | | |
| A | PSA-AF647 | Golgin97::rb-CF680 | Bleaching |
| B | VVA-AF647 | GM130::ms-CF680 | Bleaching |
| C | VEA-AF647 | Giantin::gp-CF680 | Elution, Bleaching |
| D | MAA II-AF647 | EEA1::rb-CF680 | Elution, Bleaching |
| E | CHC17-AF647 | WGA-CF680 | - |
| ***Experiment 29*** | | | |
| A | VEA-AF647 | Giantin::gp-CF680 | Bleaching |
| B | PSA-AF647 | GM130::ms-CF680 | - |
| ***Experiment 30*** | | | |
| A | VVA-AF647 | Giantin::gp-CF680 | Bleaching |
| B | PSA-AF647 | GM130::ms-CF680 | Bleaching |
| C | VEA-AF647 | Golgin97::rb-CF680 | Elution, Bleaching |
| D | MAA II-AF647 | Syntaxin6::rb-CF680 | - |
| ***Experiment 31*** | | | |
| A | DBA-AF647 | - | Bleaching |
| B | PNA-AF647 | WGA-CF680 | Bleaching |
| C | Lamin a/c::ch-AF647 | (n.r.) | Bleaching |
| D | UDA-AF647 | Fibrillarin::rb-CF680 | Bleaching |
| E | MAA II-AF647 | Con A-CF680 | Bleaching |
| F | Hoechst-JF646 | - | - |
| ***Experiment 32*** | | | |
| A | Con A-AF647 | EEA1::rb-CF680 | Bleaching |
| B | POL-AF647 | PDI::ms-CF680 | Bleaching |
| C | LCA-AF647 | LAMP5::gp-CF680 | Bleaching |
| D | PWA-AF647 | WGA-CF680 | Elution, Bleaching |
| E | UEA I-DL649 | LAMP1::ms-CF680 | Elution, Bleaching |
| F | CHC17-AF647 | Syntaxin6::rb-CF680 | Elution, Bleaching |
| G | SCAMP1::rb-AF647 | VAMP7::ms-CF680 | - |

In label names, a dash ‘-‘ symbolizes direct conjugation and a double colon ‘::’ symbolizes binding by a secondary antibody or nanobody. Abbreviations: AF647, Alexa Fluor 647; ch, chicken; gp, guinea pig; JF646, Janelia Fluor 646; ms, mouse; NB, nanobody; n.r., not relevant (if label did not work or has no relevance for this study); rb, rabbit.

## Supplementary Table 10 | Workflows for Glyco-STORM experiments in hippocampal neurons

| **Round** | **Label in channel 1** | **Label in channel 2** | **Signal removal  (post-imaging)** |
| --- | --- | --- | --- |
| ***Experiment 33*** *used in Fig. S12B, S15B, S18C* | | | |
| A | PSA-AF647 | TGN38::ms-CF680 | Bleaching |
| B | VVA-AF647 | GM130::ms-CF680 | Bleaching |
| C | VEA-AF647 | Giantin::gp-CF680 | - |
| ***Experiment 34*** | | | |
| A | PSA-AF647 | Golgin97::rb-CF680 | Bleaching |
| B | VVA-AF647 | GM130::ms-CF680 | - |
| ***Experiment 35*** | | | |
| A | PSA-AF647 | Bassoon::ms-CF680 | - |
| ***Experiment 36*** | | | |
| A | POL-AF647 | Bassoon::ms-CF680 | - |
| ***Experiment 37*** | | | |
| A | Bassoon::ms-AF647 | Con A-CF680 | - |
| ***Experiment 38*** | | | |
| A | Bassoon::ms-AF647 | WGA-CF680 | - |
| ***Experiment 39*** | | | |
| A | Homer1b/c::rb-AF647 | WGA-CF680 | - |
| ***Experiment 40*** | | | |
| A | Con A-AF647 | Homer1b/c::rb-CF680 | - |
| B | Bassoon::ms-AF647 | - |  |
| ***Experiment 41*** *used in Fig.4F,H, Fig. S17* | | | |
| A | PSA-AF647 | Homer1b/c::rb-CF680 | - |
| B | Bassoon::ms-AF647 |  |  |
| ***Experiment 42*** | | | |
| A | POL-AF647 | Homer1b/c::rb-CF680 | - |
| ***Experiment 43*** | | | |
| A | Homer1b/c::rb-AF647 | Con A-CF680 | - |
| ***Experiment 44*** | | | |
| A | POL-AF647 | (n.r.) | Elution, Bleaching |
| B | Bassoon::ms-AF647 | Homer1b/c::rb-CF680 | Elution, Bleaching |
| C | (n.r.) | WGA-CF680 | Elution, Bleaching |
| D | POL-AF647 | - | - |
| ***Experiment 45*** *used in Fig.4F* | | | |
| A | POL-AF647 | (n.r.) | Elution, Bleaching |
| B | Bassoon::ms-AF647 | Homer1b/c::rb-CF680 | Elution, Bleaching |
| C | (n.r.) | WGA-CF680 | - |
| ***Experiment 46*** *used in Fig. 4F* | | | |
| A | PSA-AF647 | (n.r.) | Elution, Bleaching |
| B | Bassoon::ms-AF647 | Homer1b/c::rb-CF680 | - |
| ***Experiment 47*** *used in Fig. 4F,H, Fig. S17* | | | |
| A | PSA-AF647 | (n.r.) | Elution, Bleaching |
| B | Bassoon::ms-AF647 | Homer1b/c::rb-CF680 | - |
| ***Experiment 48*** *used in Fig. 4F-H, Fig. S17* | | | |
| A | Con A-AF647 | Bassoon::ms-CF680 | Bleaching |
| B | Homer1b/c::rb-AF647 | WGA-CF680 | - |
| ***Experiment 49*** *used in Fig. 4F,H-J, Fig. S17* | | | |
| A | POL-AF647 | Homer1b/c::rb-CF680 | Bleaching |
| B | Bassoon::ms-AF647 | WGA-CF680 | - |
| ***Experiment 50*** *used in Fig. 4F* | | | |
| A | POL-AF647 | Bassoon::ms-CF680 | Bleaching |
| B | Homer1b/c::rb-AF647 | WGA-CF680 | Elution, Bleaching |
| C | (n.r.) | Con A-CF680 | - |
| ***Experiment 51*** *used in Fig. 4F* | | | |
| A | POL-AF647 | Bassoon::ms-CF680 | Bleaching |
| B | Homer1b/c::rb-AF647 | WGA-CF680 | Elution, Bleaching |
| C | (n.r.) | Con A-CF680 | - |
| ***Experiment 52*** *used in Fig.4F,H, Fig. S17* | | | |
| A | Con A-AF647 | Bassoon::ms-CF680 | Bleaching |
| B | Homer1b/c::rb-AF647 | WGA-CF680 | - |
| ***Experiment 53*** *used in Fig.4F,H, Fig. S17* | | | |
| A | PSA-AF647 | (n.r.) | Bleaching |
| B | Bassoon::ms-AF647 | Homer1b/c::rb-CF680 | - |
| ***Experiment 54*** *used in Fig. 4F-J, Fig. S17* | | | |
| A | Homer1b/c::rb-AF647 | WGA-CF680 | Bleaching |
| B | PSA-AF647 | Bassoon::ms-CF680 | - |
| ***Experiment 55*** *used in Fig. 4F,H, Fig. S17* | | | |
| A | Homer1b/c::rb-AF647 | WGA-CF680 | Bleaching |
| B | POL-AF647 | Bassoon::ms-CF680 | - |

In label names, a dash ‘-‘ symbolizes direct conjugation and a double colon ‘::’ symbolizes binding by a secondary antibody or nanobody. Abbreviations: AF647, Alexa Fluor 647; gp, guinea pig; ms, mouse; NB, nanobody; n.r., not relevant (if label did not work or has no relevance for this study); rb, rabbit.

## Supplementary Table References

[1] S. Sueyoshi, T. Tsuji, T. Osawa, "Purification and characterization of four isolectins of mushroom (agaricus bisporus)," *Biol. Chem. Hoppe. Seyler.* **1985**, *366*, 213.

[2] D. Bojar, L. Meche, G. Meng, W. Eng, D. F. Smith, R. D. Cummings, L. K. Mahal, "A Useful Guide to Lectin Binding: Machine-Learning Directed Annotation of 57 Unique Lectin Specificities," *ACS Chem. Biol.* **2022**, *17*, 2993.

[3] J. Kolberg, T. E. Michaelsen, K. Sletten, "Properties of a Lectin Purified from the Seeds of Cicer arietinum," *Hoppe Seylers Z Physiol Chem* **1983**, *364*, 655.

[4] A. K. Gautam, N. Gupta, D. T. Narvekar, R. Bhadkariya, S. S. Bhagyawant, "Characterization of chickpea (Cicer arietinum L.) lectin for biological activity," *Physiol. Mol. Biol. Plants* **2018**, *24*, 389.

[5] RCSB PDB - 3CNA: STRUCTURE OF CONCANAVALIN A AT 2.4 ANGSTROMS RESOLUTION, .

[6] THE STRUCTURE OF THE DOLICHOS BIFLORUS SEED LECTIN IN COMPLEX WITH THE FORSSMAN DISACCHARIDE, .

[7] A. M. Wu, A. Dudek, Y. L. Chen, "Recognition factors of Dolichos biflorus agglutinin (DBA) and their accommodation sites," *Glycoconj. J.* **2023**, *40*, 383.

[8] M. E. Etzler, E. A. Kabat, "Purification and characterization of a lectin (plant hemagglutinin) with blood group A specificity from Dolichos biflorus," *Biochemistry* **1970**, *9*, 869.

[9] E. J. M. Van Damme, W. J. Peumans, A. Barre, P. Rougé, "Plant Lectins: A Composite of Several Distinct Families of Structurally and Evolutionary Related Proteins with Diverse Biological Roles," *CRC. Crit. Rev. Plant Sci.* **1998**, *17*, 575.

[10] RCSB PDB - 2EIG: Lotus tetragonolobus seed lectin (Isoform), .

[11] T. Kawaguchi, I. Matsumoto, T. Osawa, "Studies on hemagglutinins from Maackia amurensis seeds," *J. Biol. Chem.* **1974**, *249*, 2786.

[12] Y. Yang, H. L. Xu, Z. T. Zhang, J. J. Liu, W. W. Li, H. Ming, J. K. Bao, "Characterization, molecular cloning, and in silico analysis of a novel mannose-binding lectin from Polygonatum odoratum (Mill.) with anti-HSV-II and apoptosis-inducing activities," *Phytomedicine* **2011**, *18*, 748.

[13] RCSB PDB - 2LTN: DESIGN, EXPRESSION, AND CRYSTALLIZATION OF RECOMBINANT LECTIN FROM THE GARDEN PEA (PISUM SATIVUM), .

[14] T. B. Ng, Y. S. Chan, C. C. W. Ng, J. H. Wong, "Purification and Characterization of a Lectin from Green Split Peas (Pisum sativum)," *Appl. Biochem. Biotechnol.* **2015**, *177*, 1374.

[15] I. S. Trowbridge, "Isolation and chemical characterization of a mitogenic lectin from Pisum sativum," *J. Biol. Chem.* **1974**, *249*, 6004.

[16] E. Ahmad, S. Kamranur Rahman, J. Masood Khan, A. Varshney, R. Hasan Khan, "Phytolacca americana lectin (Pa-2; pokeweed mitogen): an intrinsically unordered protein and its conversion into partial order at low pH.," *Biosci. Rep.* **2010**, *30*, 125.

[17] A. Heiskanen, T. Hirvonen, H. Salo, U. Impola, A. Olonen, A. Laitinen, S. Tiitinen, S. Natunen, O. Aitio, H. Miller-Podraza, M. Wuhrer, A. M. Deelder, J. Natunen, J. Laine, P. Lehenkari, J. Saarinen, T. Satomaa, L. Valmu, "Glycomics of bone marrow-derived mesenchymal stem cells can be used to evaluate their cellular differentiation stage," *Glycoconj. J.* **2009**, *26*, 367.

[18] J. J. Beintema, W. J. Peumans, "The primary structure of stinging nettle (Urtica dioica) agglutinin A two-domain member of the hevein family," *FEBS Lett.* **1992**, *299*, 131.

[19] R. G. Frost, R. W. Reitherman, A. L. Miller, J. S. O’Brien, "Purification of Ulex europeus hemagglutinin I by affinity chromatography," **1975**, *69*, 170.

[20] N. Fornstedt, J. Porath, "Characterization studies on a new lectin found in seeds of Vicia ervilia," *FEBS Lett.* **1975**, *57*, 187.

[21] I. J. Goldstein, R. D. Poretz, in *The Lectins*, Elsevier **1986**, pp. 33–247.

[22] C. S. Wright, "Crystallographic elucidation of the saccharide binding mode in wheat germ agglutinin and its biological significance," *J. Mol. Biol.* **1980**, *141*, 267.
